# Autophagy regulates cellular senescence by mediating the degradation of CDKN1A/p21 and CDKN2A/p16 through SQSTM1/p62-mediated selective autophagy in myxomatous mitral valve degeneration

**DOI:** 10.1080/15548627.2025.2469315

**Published:** 2025-03-04

**Authors:** Qiyu Tang, Keyi Tang, Greg R. Markby, Maciej Parys, Kanchan Phadwal, Vicky E MacRae, Brendan M. Corcoran

**Affiliations:** a The Roslin Institute, The University of Edinburgh, Edinburgh, UK; b Royal (Dick) School of Veterinary Studies, The University of Edinburgh, Edinburgh, UK; c School of Life Sciences, Faculty of Science and Engineering, Anglia Ruskin University, Cambridge, UK

**Keywords:** Autophagic degradation, CDKI, MMVD, SASP, TGFB, ubiquitin-proteasome pathway

## Abstract

Myxomatous mitral valve degeneration (MMVD) is one of the most important age-dependent degenerative heart valve disorders in both humans and dogs. It is characterized by the aberrant remodeling of extracellular matrix (ECM), regulated by senescent myofibroblasts (aVICs) transitioning from quiescent valve interstitial cells (qVICs), primarily under TGFB1/TGF-β1 control. In the present study, we found senescent aVICs exhibited impaired macroautophagy/autophagy as evidenced by compromised autophagy flux and immature autophagosomes. MTOR-dependent autophagy induced by rapamycin and torin-1 attenuated cell senescence and decreased the expression of cyclin-dependent kinase inhibitors (CDKIs) CDKN2A/p16^INK4A^ and CDKN1A/p21^CIP1^. Furthermore, induction of autophagy in aVICs by *ATG* (autophagy related) gene overexpression restored autophagy flux, with a concomitant reduction in CDKN1A and CDKN2A expression and senescence-associated secretory phenotype (SASP). Conversely, autophagy deficiency induced CDKN1A and CDKN2A accumulation and SASP, whereas ATG re-expression alleviated senescent phenotypic transformation. Notably, CDKN1A and CDKN2A localized to autophagosomes and lysosomes following MTOR antagonism or MG132 treatment. SQSTM1/p62 was identified as the autophagy receptor to selectively sequester CDKN1A and CDKN2A cargoes for autophagic degradation. Our findings are the first demonstration that CDKN1A and CDKN2A are degraded through SQSTM1-mediated selective autophagy, independent of the ubiquitin-proteasome pathway. These data will inform development of therapeutic strategies for the treatment of canine and human MMVD, and for the treatment of Alzheimer disease, Parkinson disease and other age-related degenerative disorders.

**Abbreviations**: ACTA2/α-SMA: actin alpha 2, smooth muscle; AKT: AKT serine/threonine kinase; aVICs: activated valve interstitial cells; ATG: autophagy related; baf-A1: bafilomycin A_1_; BrdU, bromodeoxyuridine; BSA: bovine serum albumin; CDKIs, cyclin-dependent kinase inhibitors; CDKN1A/p21: cyclin dependent kinase inhibitor 1A; CDKN2A/p16: cyclin dependent kinase inhibitor 2A; co-IP: co-immunoprecipitation; DMSO: dimethylsulfoxide; ECM, extracellular matrix; EIF4EBP1: eukaryotic translation initiation factor 4E binding protein 1; eGFP: green fluorescent protein; ELISA: enzyme-linked immunosorbent assay; HEK-293T, human embryonic kidney 293T; HRP: horseradish peroxidase; KO: knockout; MAP1LC3/LC3: microtubule associated protein 1 light chain 3; LIR: MAP1LC3/LC3-interacting region; MFS: Marfan syndrome; MKI67/Ki-67: marker of proliferation Ki-67; MMVD: myxomatous mitral valve degeneration; MTOR: mechanistic target of rapamycin kinase; MTORC: MTOR complex; OE: overexpression; PBST, phosphate-buffered saline with 0.1% Tween-20; PCNA: proliferating cell nuclear antigen; PIK3CA/PI3K: phosphatidylinositol-4,5-bisphosphate 3-kinase catalytic subunit alpha; PLA: proximity ligation assays; PSMA1: proteasome 20S subunit alpha 1; PSMB5: proteasome 20S subunit beta 5; qVICs: quiescent valve interstitial cells; qRT-PCR: quantitative real-time PCR; SA-GLB1/β-gal: SA-senescence-associated GLB1/β-galactosidase; ROS: reactive oxygen species; SASP: senescence-associated secretory phenotype; RPS6KB1/p70 S6K: ribosomal protein S6 kinase B1; SMAD: SMAD family member; SQSTM1/p62: sequestosome 1; STEM: scanning transmission electron microscopy; TGFB: transforming growth factor beta; TGFBR: transforming growth factor beta receptor; TP53/p53: tumor protein p53; UPS: ubiquitin-proteasome system; WT, wild-type.

## Introduction

Myxomatous mitral valve degeneration (MMVD), also referred to as mitral valve prolapse (MVP), is one of most important degenerative cardiovascular diseases associated with aging in both dogs and humans. This disorder is a leading contributor to cardiac failure and death, resulting in substantial morbidity and mortality [1–4]. MMVD is responsible for approximately 7% of canine fatalities before the age of 10 and its occurrence in older dogs ranges from 30% to 70% [1,5]. In humans MMVD affects 2%-3% of the worldwide population. Fifteen percent of the affected patients require surgical intervention for valve repair or replacement, which are complex and expensive, posing heightened risks for older adults and potentially leading to severe consequences such as thrombosis and myocardial infarction [6,7]. Currently, there are no therapeutic agents capable of preventing, decelerating progression or reversing the valve deterioration linked with MMVD. Therefore, a deeper insight into the mechanisms underlying MMVD’s development is necessary if innovative treatment approaches for this condition in both humans and canines are to be developed.

The progression of mitral valve degeneration involves aberrant extracellular matrix (ECM) remodeling (myxomatous degeneration) and is primarily controlled by TGFB (transforming growth factor beta) [4,8]. There is growing clinical and experimental evidence that TGFB participates in the fibrotic and myxomatous changes seen in end-stage MMVD in humans [9–14]. Elevated activity of TGFB has been identified in diverse MVP forms including those affected by Barlow’s Disease and Marfan syndrome (MFS). Pathological conditions that cause increased circulating TGFB levels have been correlated with myxomatous valvulopathies in MFS [4]. Moreover, TGFB signaling is notably augmented in a genetically modified FBN1 (fibrillin 1)-deficient murine model of MFS [14]. This is further confirmed by surgical specimens of myxomatous valves from patients with advanced disease, demonstrating pronounced TGFB expression across all valvular layers [13]. Comparable findings are evident in cases of spontaneously occurring MMVD in dogs [15,16].

A significant expansion of activated myofibroblast phenotypes (aVICs), which originate from quiescent valve interstitial cells (qVICs) and are characterized by the expression of ACTA2/α-SMA (actin alpha 2, smooth muscle), has been observed in myxomatous mitral valves in both humans and canines [17,18]. TGFB has been shown to prompt this aberrant transdifferentiation of aVICs and thereby promote increased production of excessive ECM [11,19,20]. The pharmacological inhibition of TGFBR (transforming growth factor beta receptor) reverts aVICs to a normal, quiescent state [19]. The role of the downstream canonical SMAD2 (SMAD family member 2)-SMAD3 pathway in TGFB signaling has been shown to control VIC phenotype and ECM synthesis [11–14,18]. However, a multicenter clinical trial involving patients with MFS revealed no significant impact from inhibiting the TGFB mediated canonical SMAD2-SMAD3 cascade through the administration of the AGTR (angiotensin II receptor) blocker losartan. These findings imply that further investigation into the non-SMAD-mediated mechanisms of the TGFB signaling pathway may provide valuable insights. Additionally, exploring the non-canonical branches of this pathway could offer advantageous perspectives [21]. Notably, PIK3CA/PI3K (phosphatidylinositol-4,5-bisphosphate 3-kinase catalytic subunit alpha)-AKT (AKT serine/threonine kinase)-MTOR (mechanistic target of rapamycin kinase) pathway garners significant interest due to its association with a spectrum of age-related degenerative conditions, encompassing osteoarthritis, atherosclerosis, and thrombosis [22,23]. Recently, we identified that myxomatous remodeling in mitral valves is likely regulated by the TGFB downstream PI3K-AKT-MTOR pathway, using an *in vitr*o naturally occurring early stage canine model of MMVD [24]. This canine model approximates the pre-fibrotic stage of human MMVD. Activation of PI3K-AKT-MTOR signaling induced by TGFB1 was found to transition healthy qVICs into senescent aVIC phenotypes. These senescent VICs exhibited characteristics of proliferation arrest, CDKN1A-TP53/p53 (tumor protein p53) and CDKN2A activation and reduced autophagy. Interestingly, though in a growth arrested state, these senescent aVICs remain metabolically active and demonstrate significant changes in their secretome, with an increased senescence associated secretory phenotype (SASP). Within this secretory phenotype, senescent aVICs excrete a multifaceted cocktail of factors, notably including TGFBs. This secretion perpetuates ongoing aberrant ECM remodeling. Additionally, these cells exhibit resistance to tissue removal through cellular apoptosis [24].

Autophagy is an evolutionarily conserved catabolic cellular process by which cytoplasmic proteins and organelles are engulfed within autophagosomes and degraded after fusion with lysosomes [25]. It is regulated by essential conserved *ATG* (autophagy related) genes and plays a critical role in maintaining cellular homeostasis. Additionally, impairment of autophagy has been linked to multiple age-related degenerative diseases including Alzheimer disease and Parkinson disease [26,27]. Autophagy deficiency promotes senescence-associated microglia as evidenced by reduced proliferation, increased CDKN1A and senescence-associated secretory phenotype in Alzheimer disease [28]. Furthermore, inhibition of autophagy flux induces cell senescence in primary human fibroblasts and murine NIH3T3 fibroblasts [29,30]. It seems likely that disruption of autophagy may contribute to the aberrant transformation of VICs to an active senescent phenotype and so contribute to MMVD pathogenesis. Given the pivotal role of autophagy in various degenerative diseases, coupled with insights from our preliminary research, we posit that an investigation into how autophagy modulates senescence in this disease context would be of substantial benefit.

Interest in examining canine diseases as models of age-related diseases in humans has emerged in recent years [3,4,24,31–36]. Undoubtedly, the exploration of the MMVD pathogenesis in canines is indisputably instrumental in enhancing our comprehension of analogous disease processes in humans. Research on human MMVD has predominantly relied on analysis of surgically excised samples derived from patients with advanced-stage disease and transgenic mouse models. While mice with genetic modifications provide significant understanding into various molecular events, their limitations lie in accurately replicating the tri-layered human valvular structure, the chronic nature of the disease, and their inability to produce the pathology of myxomatous degeneration in mitral valves. Moreover, human samples derived from end-stage disease often exhibit extensive TGFB-induced fibrosis, which complicates the study of molecular mechanisms driving the initial development of pre-fibrosis myxomatous alterations mediated by TGFB [4]. Canines, possessing a similar tri-layer valve structure but devoid of end-stage fibrosis, share pathophysiological and molecular traits with human MMVD, permitting investigation as the disease emerges and progresses. Investigating cellular and molecular events in canines can yield valuable insights applicable to both species, thereby enriching both veterinary and human medical fields.

In the present study we report that senescent cells, derived from dogs with characterized early to mid-stage MMVD, exhibit deficient autophagy flux and autophagosome maturation. We have found that the MTOR dependent antagonist rapamycin and torin-1 decreases CDKN2A and CDKN1A expression and alleviates cell senescence. We have further demonstrated that autophagy promotion attenuates cell senescence whereas autophagy deficiency induces a senescence-associated phenotype with increased expression of SASP. Notably, we identify a novel degradation pathway for cyclin-dependent kinase inhibitors (CDKIs) CDKN2A and CDKN1A, which occurs through SQSTM1-mediated selective autophagy and operates independently of the ubiquitin-proteasome system (UPS). Our research elucidates the protective function of autophagy in VICs, highlighting its regulatory impact on the progression of MMVD and its capacity to limit cellular senescence. These findings suggest that MTOR inhibitors might possess innovative therapeutic potential for the management of MMVD, as well as other age-related degenerative disorders, in affected canines and by extension in human patients.

## Results

### Autophagy flux is impaired in senescent aVICs

**Figure 1.**
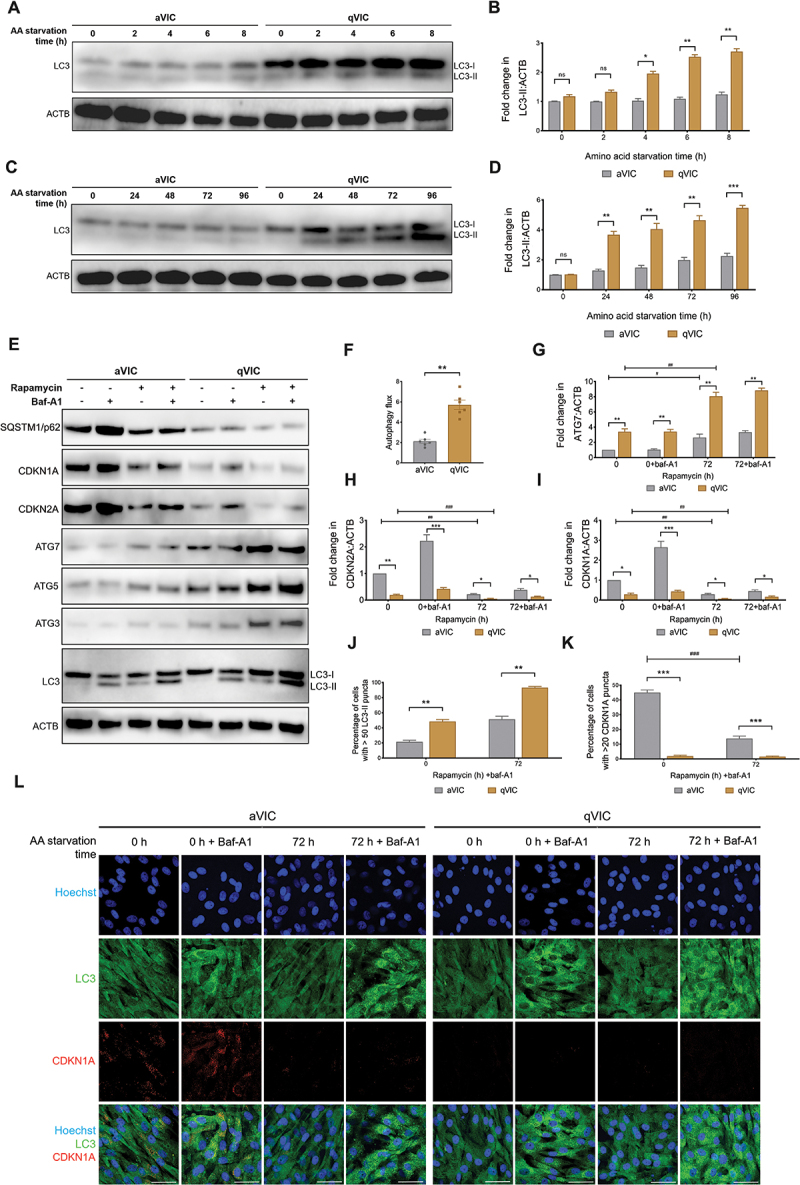
Senescent aVICs exhibit impaired autophagy flux. (A and C) Representative images of MAP1LC3/LC3 and ACTB (loading control) immunoblots of aVICs and qVICs following amino acid starvation for the indicated time periods (n = 3). (B and D) MAP1LC3/LC3-II:ACTB ratio normalized to 0 h for aVICs and qVICs treated as in (A and B). (E) Representative images of immunoblots in aVICs and qVICs treated with or without 200 nM rapamycin for 48 h in the presence or absence of 10 µM baf-A1 for 4 h (n = 4). (F) Ratio of (MAP1LC3/LC3-II + baf-A1/ACTB):(MAP1LC3/LC3-ii + baf-A1/ACTB following 48 h rapamycin treatment), corresponding to MAP1LC3/LC3-II turnover (n = 6). (G-I) Ratio of ATG7 (G), CDKN2A/p16 (H), CDKN1A/p21 (I) to ACTB in cells, normalized to aVICs in the absence of rapamycin and baf-A1, that were treated with or without 200 nM rapamycin and 10 µm baf-A1 as in (E). (J) Percentage of aVICs and qVICs (treated with or without AA starvation and baf-A1) with > 50 MAP1LC3/LC3-II puncta as shown in (L). (K) Percentage of aVICs and qVICs (treated with or without AA starvation and baf-A1) with > 20 CDKN1A/p21 puncta as shown in (L). (L) Representative images of MAP1LC3/LC3 and CDKN1A/p21 immunostaining in aVICs and qVICs treated with or without amino acid deprivation for 72 h in the presence or absence of 10 µM baf-A1 for 4 h (n = 3). Scale bars: 50 µm. These experiments were repeated at least three times (n ≥ 3), and results are presented as mean ± SEM. ANOVA followed by Tukey’s range test. (ns, not significant; *p < 0.05; **p < 0.01; ***p < 0.001; ^#^*p* <0.05; ^##^*p* <0.01; ^###^*p* <0.001).

Our recent study showed that PI3K-AKT-MTOR signaling is activated in aVICs. These cells are in a senescent state with impaired autophagy, as evidenced by decreased expression of ATG7 in CDKN1A positive aVICs in myxomatous mitral valve tissues [24]. In the present study, in order to further investigate the autophagy state in MMVD, autophagy was examined in qVICs and aVICs following amino acid withdrawal, an established method of autophagy induction [37]. The protein expression levels of the autophagosome marker MAP1LC3/LC3 (microtubule associated protein 1 light chain 3)-II were significantly reduced both during short-term (Figure 1A,B) and prolonged starvation in aVICs compared with qVICs (Figure 1C,D). Furthermore, the baseline level of autophagy flux was assessed in qVICs and aVICs. Inhibition of MAP1LC3 degradation in autolysosomes with bafilomycin A_1_ (baf-A1) revealed that autophagy flux became markedly reduced in aVICs compared with qVICs in the presence or absence of the autophagy inducer rapamycin [38] (Figure 1E,F). The expression of key regulatory autophagic genes *ATG3*, *ATG5* and *ATG7*, which control autophagosome formation by regulating the conversion MAP1LC3-I to MAP1LC3-II, were markedly decreased in aVICs, both in the absence or presence of rapamycin (Figure 1E,G). SQSTM1, known to accumulate in cells with diminished autophagic flux [39], exhibited heightened levels in senescent aVICs while decreased in the presence of rapamycin (Figure 1E). Notably, CDKN1A and CDKN2A were markedly decreased both in qVICs and aVICs under conditions of autophagy induction by rapamycin (Figure 1E,H,I). MAP1LC3 immunostaining confirmed the levels of MAP1LC3-II were significantly elevated in qVICs compared with aVICs during prolonged amino acid deprivation (Figure 1J,L). CDKN1A were significantly reduced in aVICs after amino acid withdrawal (Figure 1K,L).

Cellular senescence is recognized as a significant contributor to autophagy dysfunction [30]. To explore whether the proteins CDKN2A and CDKN1A are sufficient to induce a senescent phenotype in VICs, CDKN2A and CDKN1A were overexpressed in qVICs. Our results revealed that the overexpression of CDKN2A and CDKN1A led to the accumulation of CDKN2A, CDKN1A and TP53 in VICs (Fig. S1A), which was associated with a reduction in the expression of PCNA (proliferating cell nuclear antigen) and MKI67/Ki-67 (marker of proliferation Ki-67), along with decreased incorporation of bromodeoxyuridine (BrdU) (Fig. S1B-S1D), indicative of diminished cellular proliferation. Concurrently, there was a notable increase in senescence-associated GLB1/β-galactosidase (SA-GLB1/β-gal) activity (Fig. S1E), reinforcing the emergence of a senescent phenotype. Furthermore, a downregulation of ATG3, ATG5 and ATG7, accompanied by an elevation in SQSTM1 levels, was observed, along with a reduced ratio of MAP1LC3-II to I, indicating an impairment in autophagic flux (Fig. S1F-S1H). Confocal microscopy further substantiated the significant reduction in ATG7 levels in cells overexpressing CDKN2A and CDKN1A (Fig. S1I). Collectively, these findings imply that CDKN2A and CDKN1A overexpression (OE) induced a senescent phenotype in VICs with a compromised autophagic flux.

### Autophagosome maturation is compromised in senescent aVICs

**Figure 2.**
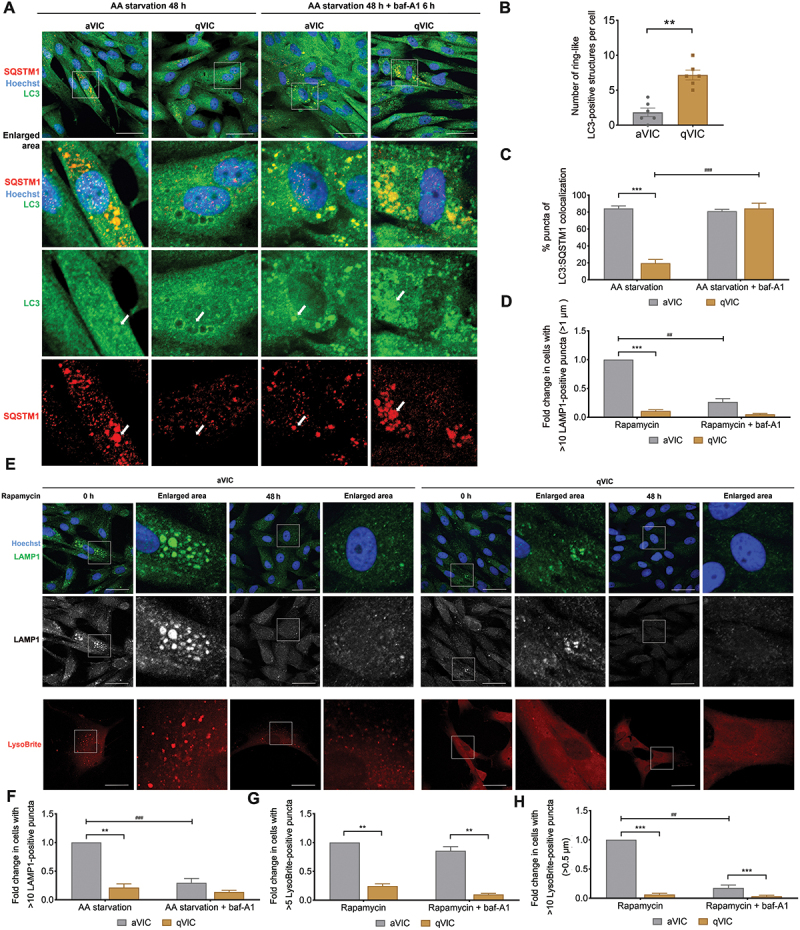
Senescent aVICs show immature autophagosomes. (A) Representative confocal images of MAP1LC3/LC3 and SQSTM1/p62 immunostaining in aVICs and qVICs in full medium and following amino acid starvation for 48 h in the presence or absence of 10 µm baf-A1 for 6 h. The white arrow locates MAP1LC3/LC3-positive ring-like structures. Scale bars: 50 µm. (B) Quantification of MAP1LC3/LC3-positive ring-like structures per cell in VICs following amino acid deprivation for 48 h as in (A) (n = 6). (C) Quantification of MAP1LC3/LC3 colocalizing with SQSTM1/p62 puncta in VICs as described in (A) (n = 3). (D) Quantification of VICs (%) with > 10 LAMP1 puncta (>1 µm) in the presence of 200 nM rapamycin with or without baf-A1 as shown in (E). (E) Representative images (top) of LAMP1 immunostaining in aVICs and qVICs treated with or without 200 nM rapamycin for 48 h (n = 5). Scale bars: 50 µm. Representative images (bottom) of LysoBrite staining in VICs in the presence or absence of 200 nM rapamycin for 48 h. Scale bars: 50 µm (n = 3). (F) Quantification of VICs (%) with > 10 LAMP1 puncta in the presence of 200 nM rapamycin with or without baf-A1as shown in (E). (G) Quantification of VICs (%) with > 5 LysoBrite puncta in the presence of 200 nM rapamycin with or without baf-A1as shown in (E). (H) Quantification of VICs (%) with > 10 LysoBrite puncta (>0.5 µm) in the presence of 200 nM rapamycin with or without baf-A1as shown in (E). These experiments were repeated at least three times (n ≥ 3), and results are presented as mean ± SEM. ANOVA followed by Tukey’s range test. (ns, not significant; *p < 0.05; **p < 0.01; ***p < 0.001; ^#^*p* <0.05; ^##^*p* <0.01; ^###^*p* <0.001).

MAP1LC3-positive vesicular formations in qVICs during prolonged amino acid deprivation coalesced into predominantly perinuclear, ring-shaped aggregates (Figure 2A,B), previously identified as transitional entities in autophagosome maturation [40]. In contrast, these structures were sporadic, with smaller MAP1LC3-positive vesicles prevailing instead in aVICs (Figure 2A,C). Furthermore, the autophagy receptor SQSTM1, typically degraded in autolysosomes along with its cargo [41], showed an intriguing pattern: ring-like structures in qVICs infrequently colocalized with SQSTM1, whereas smaller MAP1LC3-positive vesicles in aVICs frequently demonstrated SQSTM1 colocalization (Figure 2A,C). These observations imply altered cargo recognition and sequestration in aVICs, given the established role of SQSTM1 in directing cargo to phagophores (the precursors to autophagosomes) and its colocalization with MAP1LC3 [41]. The sustained SQSTM1-MAP1LC3 colocalization in aVICs may suggest that the impaired degradation of SQSTM1 is potentially due to either disrupted fusion between autophagosome and lysosome or diminished acidity within autolysosomes. Application of baf-A1, an inhibitor of the fusion between autophagosomes and lysosomes, reinstated SQSTM1 and MAP1LC3 colocalization in qVICs but was ineffectual in aVICs (Figure 2A,C). These findings suggest that the annular SQSTM1-deficient structures observed in qVICs presumably signify mature autolysosomes containing partially digested cytoplasmic content.

To further elucidate the process of autophagosome maturation in VICs, the fusion between autophagosomes and lysosomes was assessed using VICs expressing the tandem mCherry-enhanced green fluorescent protein (eGFP)-MAP1LC3B/LC3B construct (Fig. S2A). This construct allows the visualization of autophagosome (yellow) and autolysosomes (red), due to the quenching of eGFP fluorescence in the acidic lysosomal environment [39]. A reduced proportion of autolysosomes was detected in aVICs compared to qVICs, both in the presence and absence of autophagy inducers rapamycin or torin-1 (Fig. S2A and S2B), suggesting an impairment in autophagosome maturation in aVICs. Additionally, a higher number of autophagosomes and autolysosomes was observed in qVICs compared to aVICs, regardless of rapamycin or torin-1 treatment (Fig. S2A and S2C). Consistent with these findings, scanning transmission electron microscopy (STEM) confirmed that qVICs exhibited a greater abundance of autophagosomes and autolysosomes than aVICs both in the absence and presence of rapamycin or torin-1 (Fig. S2D and S2E).

Assessment of senescent aVICs revealed that they contained increased lysosomal size (Figure 2D,E) and number (Figure 2E,F) compared with qVICs in the presence or absence of rapamycin. LysoBrite staining of acidic vesicles in lysosomes confirmed these observations (Figure 2E,G,H), indicating lysosomal dysfunction in aVICs. Taken together, these results suggest that autophagosome maturation is an important characteristic of senescent aVICs.

### Rapamycin reverses cell senescence and promotes autophagy

**Figure 3.**
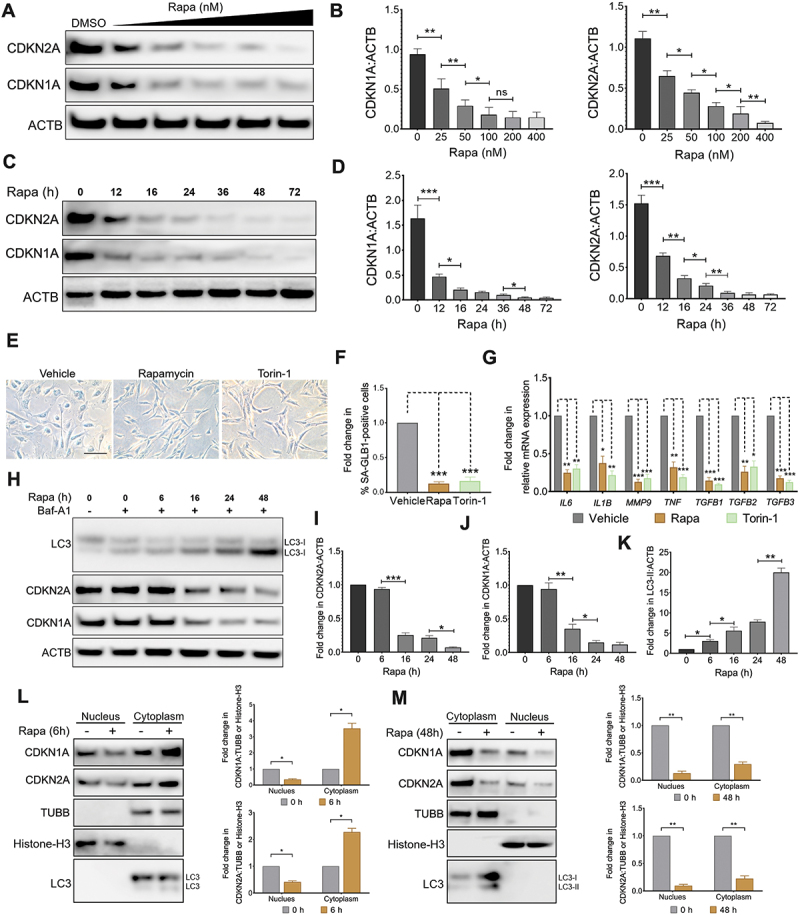
Rapamycin reverses cell senescence and improves autophagy. (A) Representative images of CDKN1A/p21, CDKN2A/p16 and ACTB (loading control) immunoblots of aVICs treated with the indicated dose ranges of rapamycin (Rapa, 25, 50, 100, 200, or 300 nM) for 48 h (n = 3). (B) CDKN1A/p21 (left panel) and CDKN2A/p16 (right panel):actb ratio in cells, normalized to aVICs in the absence of rapamycin, that were treated as in (A). (C) Representative images of CDKN1A/p21, CDKN2A/p16 and ACTB immunoblots of aVICs treated with rapamycin for the indicated time periods (n = 3). (D) CDKN1A/p21 (left panel) and CDKN2A/p16 (right panel):actb ratio in cells, normalized to 0 h for aVICs treated as in (C). (E) Representative images of SA-GLB1/β-gal staining in aVICs treated with either 200 nM rapamycin for 72 h or 80 nM torin-1 for 48 h. Scale bars: 100 µm (n = 3). (F) Percentage of SA-GLB1/β-gal-positive aVICs treated with either rapamycin or torin-1 as shown in (E). (G) Quantification of SASP cytokine expression by qRT-pcr in aVIC cultures treated with either rapamycin or torin-1 (n = 4). (H) Representative images of CDKN1A/p21, CDKN2A/p16, MAP1LC3/LC3 and ACTB immunoblots of aVICs treated with rapamycin for the indicated time periods in the absence or presence of 5 µm baf-A1 for 4 h (n = 3). (I-K) Quantification of CDKN1A/p21 (I), CDKN2A/p16: (J) and MAP1LC3/LC3: (K) ACTB ratio in cells, normalized to 0 h for aVICs in the presence of baf-A1, that were treated as in (H). (L and M) Representative images (left panel) of immunoblots for the indicated proteins in the cytoplasm and nucleus fractions isolated from aVICs treated with 200 nM rapamycin for 6 h (L) and 48 h (M) (n = 3). The graphs (right panel) show the ratios from the densitometry analysis of the displayed experiment for each protein to TUBB/β-tubulin (cytoplasmic fractions) or histone-H3 (nuclear fractions) normalized to the corresponding aVICs in the absence of rapamycin. These experiments were repeated at least three times (n ≥ 3), and results are presented as mean ± SEM. ANOVA followed by Tukey’s range test. (ns, not significant; *p < 0.05; **p < 0.01; ***p < 0.001; ^#^*p* <0.05; ^##^*p* <0.01; ^###^*p* <0.001).

We have previously shown that silencing RPS6KB1/p70 S6K (ribosomal protein S6 kinase B1), the key functional transcription factor of MTOR, attenuates cell senescence and promotes autophagy flux in aVICs [24]. To substantiate this observation, we subsequently assessed the expression levels of CDKN1A and CDKN2A in aVICs following treatment with two MTOR inhibitors: rapamycin, which predominantly inhibits MTOR complex 1 (MTORC1), and torin-1, a potent inhibitor of both MTORC1 and MTORC2 [42]. Rapamycin treatment led to a dose- (Figure 3A,B) and time-dependent (Figure 3C,D) reduction in CDKN2A and CDKN1A expression. A comparable dose- and time-dependent effect was observed with torin-1 (Fig. S3A and S3B). To examine the impact of these inhibitors on MTOR activity in aVICs, the phosphorylation of RPS6KB1 at Ser389 (p-RPS6KB1), EIF4EBP1 (eukaryotic translation initiation factor 4E binding protein 1) at Ser65, Thr37 and Thr46 (p-EIF4EBP1), and AKT at Ser473 (p-AKT) was analyzed. Both treatments decreased p-RPS6KB1 and p-EIF4EBP1 levels, confirming MTORC1 inhibition (Fig. S3C and S3D). Additionally, torin-1 treatment reduced AKT phosphorylation at Ser 473, indicating MTORC2 inhibition (Fig. S3C and S3D). Interestingly, rapamycin treatment led to an increase in AKT phosphorylation at Ser 473, potentially attributable to the well-established negative feedback loop between RPS6KB1 and AKT within the IRS1 (insulin receptor substrate 1)-PI3K-AKT-MTOR signaling pathway [43]. This pathway has been known to be activated in canine MMVD and other pathophysiological conditions [24,44], suggesting a possible mechanistic link.

To further examine the impact of rapamycin and torin-1 on cell senescence, the expression profile of senescent markers were assessed in aVICs. Treatment with both inhibitors decreased SA-GLB1 activity (Figure 3E,F), reactive oxygen species (ROS) levels (Fig. S3E), and reduced the expression of SASP components (Figure 3G) in aVICs. Interestingly, cells treated with rapamycin demonstrated increased BrdU incorporation along with elevated expression of MKI67 and PCNA (Fig. S3F-S3J), indicating enhanced cell proliferation while this pro-proliferative effect was absent in cells treated with torin-1 (Fig. S3F-S3J). Moreover, the rapamycin-induced proliferation could be abolished following torin-1 treatment for 48 h, as evidenced by reduced MKI67 and PCNA protein expression and diminished BrdU incorporation (Fig. S3G-S3J).

Moreover, treatment with (i) rapamycin, (ii) torin-1, or (iii) both inhibitors in combination significantly decreased ULK1 (unc-51 like autophagy activating kinase 1) phosphorylation at Ser757 (Fig. S3G and S3H), a key regulator of ULK1 activation and autophagy initiation [45]. We found the protein expression levels of both CDKN2A and CDKN1A were comparable after 6 h rapamycin exposure but became markedly reduced along with increased MAP1LC3-II expression after 48 h treatment (Figure 3H-K). Similarly, the protein expression levels of CDKN2A and CDKN1A were decreased, accompanied by an enhancement in autophagy following torin-1 treatment for 48 h, both in the presence or absence of baf-A1 (Fig. S4A-S4C). Since CDKN2A and CDKN1A, as the main members of CDKIs, can be found distributed both in the nucleus and cytoplasm in response to various signals [46]. Next, we explored the impact of rapamycin on the subcellular expressions of both proteins. A nuclear and cytosolic fractionation assay revealed that elevated expression levels of both CDKN2A and CDKN1A in the cytoplasm and reduced expression in the nucleus were observed during short-term rapamycin treatment, whereas prolonged rapamycin treatment significantly reduced CDKN2A and CDKN1A expression in both the nuclear and cytoplasmic fractions (Figure 3L,M). These findings are consistent with our confocal microscopy results (Fig. S4D and S4E). Together, these results suggest that rapamycin reduces the protein expression of CDKN2A and CDKN1A and attenuates cell senescence in aVICs.

### ATG overexpression attenuates cell senescence in aVICs

**Figure 4.**
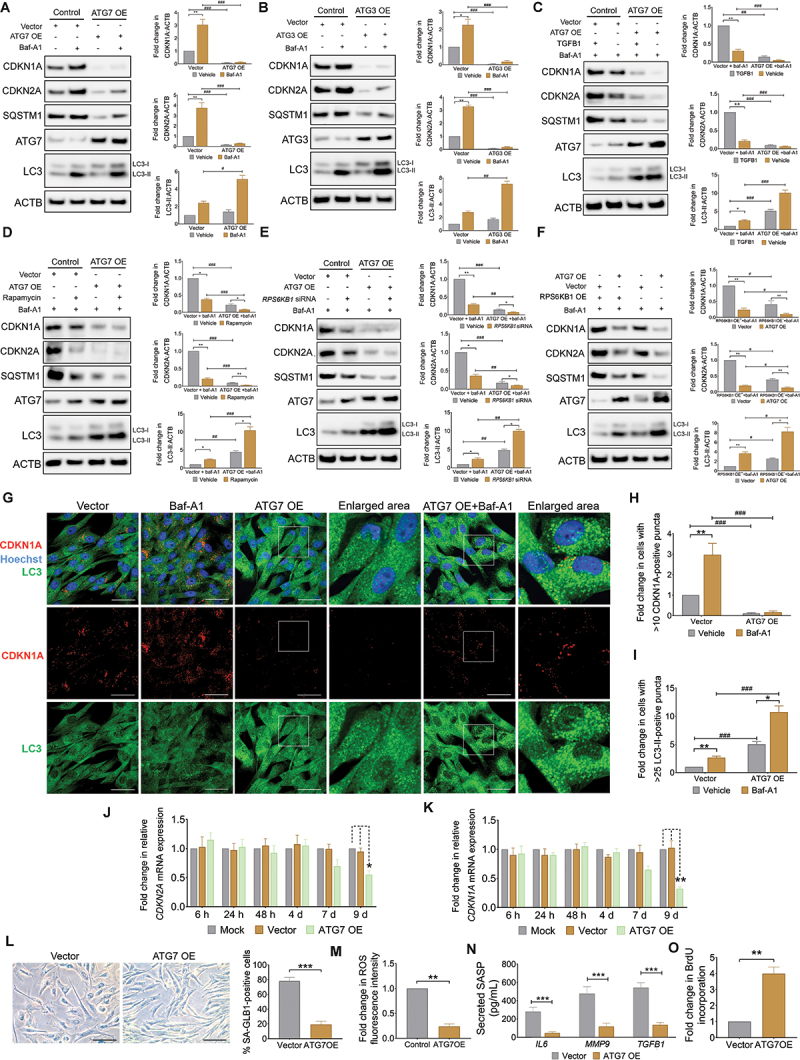
ATG overexpression alleviates aVIC senescence. (A and B) Representative images (left panel) of CDKN1A/p21, CDKN2A/p16, SQSTM1/p62, ATG7, ATG3, MAP1LC3/LC3 and ACTB (loading control) immunoblots of aVICs transfected with empty vectors, *pcDNA3.1-Flag-ATG7* (A) or *pcDNA3.1-Flag-ATG3* (B) plasmids in the presence or absence of 5 µm baf-A1 for 4 h (n = 3). The graphs (right panel) show the ratios of CDKN1A/p21, CDKN2A/p16, MAP1LC3/LC3-II to ACTB normalized to aVICs transfected with empty vectors in the absence of baf-A1. (C-E) Representative images (left panel) of CDKN1A/p21, CDKN2A/p16, SQSTM1/p62, ATG7, MAP1LC3/LC3 and ACTB (loading control) immunoblots of aVICs transfected with empty vectors or *pcDNA3.1-Flag-ATG7* plasmids and then treated with or without 10 ng/mL TGFB1 for 72 h (C), 200 nM rapamycin for 72 h (D) or *RPS6KB1*-specific siRNA for 24 h (E) in the presence of 5 µm baf-A1 for 4 h (n = 3). The graphs (right panel) show the ratios of CDKN1A/p21, CDKN2A/p16, MAP1LC3/LC3-II to ACTB normalized to aVICs transfected with empty vectors in the presence of baf-A1. (F) Representative images (left panel) of CDKN1A/p21, CDKN2A/p16, SQSTM1/p62, ATG7, MAP1LC3/LC3 and ACTB (loading control) immunoblots of HEK293T cells transfected with empty vectors or *pcDNA3.1-Flag-RPS6KB1* plasmids and then re-expressed with or without *pcDNA3.1-Flag-ATG7* plasmids in the presence of 5 µm baf-A1 for 4 h (n = 3). The graphs (right panel) show the ratios of CDKN1A/p21, CDKN2A/p16, MAP1LC3/LC3-II to ACTB normalized to aVICs transfected with empty vectors in the presence of baf-A1. (G) Representative confocal images of CDKN1A/p21 and MAP1LC3/LC3 immunostaining in aVICs transfected with empty vectors or *pcDNA3.1-Flag-ATG7* plasmids for 24 h in the absence or presence of 5 µm baf-A1 for 4 h (n = 3). Scale bars: 50 µm. (H and I) Quantification of aVICs (%) with > 10 CDKN1A/p21 puncta (H) or > 25 MAP1LC3/LC3-II puncta (I) transfected with empty vectors or *pcDNA3.1-Flag-ATG7* plasmids with or without baf-A1 as shown in (G). (J and K) Cells were transfected with either empty vectors (vector), canine *pcDNA3.1-Flag-ATG7*, or subjected to mock transfection (mock) without vectors. The mRNA expression levels of *CDKN2A/p16* (J) and *CDKN1A/p21* (K) were quantified at 6 h, 24 h, 48 h, 4 day, 7 day and 9 day post-transfection (n = 4). (L) Representative images (left panel) of SA-GLB1/β-gal staining in aVICs transfected with empty vectors or *pcDNA3.1-Flag-ATG7* plasmids. Scale bars: 100 µm (n = 3). Percentage (right panel) of SA-GLB1/β-gal positive cells. (M) Quantification of ROS fluorescence intensity for cells transfected with empty vectors or *pcDNA3.1-Flag-ATG7* plasmids (n = 3). (N) Quantification of secreted TGFB1, IL6 and MMP9 cytokine expression detected by ELISA in collected supernatant from VIC cultures transfected with empty vectors or *pcDNA3.1-Flag-ATG7* plasmids (n = 3). (O) Quantification of BrdU incorporation for aVICs transfected with empty vectors or *pcDNA3.1-Flag-ATG7* plasmids (n = 4). These experiments were repeated at least three times (n ≥ 3), and results are presented as mean ± SEM. ANOVA followed by Tukey’s range test. (ns, not significant; *p < 0.05; **p < 0.01; ***p < 0.001; ^#^*p* <0.05; ^##^*p* <0.01; ^###^*p* <0.001).

Autophagy is a dynamic cellular process that involves the degradation and recycling of damaged or unnecessary cellular components through rapid cargo transport and turnover [25]. To further investigate the role of autophagy in the degradation of CDKN2A and CDKN1A, two essential autophagy genes *ATG7* and *ATG3*, controlling autophagosome formation, were overexpressed in aVICs. Immunoblot results showed that in both the absence or presence of baf-A1 the protein expression levels of CDKN2A, CDKN1A and the autophagy cargo receptor SQSTM1 (Figure 4A,B) were significantly decreased, along with significantly increased autophagy flux in AGT7 and ATG3 OE aVICs (Figure 4A,B). Notably, the reduction of CDKN2A, CDKN1A and SQSTM1 protein expressions was partially restored by addition of TGFB1, an effective inducer of MTOR activation (Figure 4C) [24,47]. Conversely, rapamycin, and human *RPS6KB1*-specific siRNA, which induces autophagy by inactivating MTOR-RPS6KB1 signaling [24,48], significantly decreased CDKN2A, CDKN1A and SQSTM1 protein expression in aVICs (Figure 4D,E). To confirm our findings, we next examined the effects of autophagy promotion on CDKN2A and CDKN1A degradation in human embryonic kidney 293T (HEK-293T) cells. Overexpression of human ATG7 induced CDKN2A, CDKN1A and SQSTM1 removal, and this effect was partially attenuated in HEK293T cells following human RPS6KB1 overexpression (Figure 4F). Consistent with these findings, overexpression of ATG7 led to a decrease in CDKN1A levels, accompanied by an elevation in MAP1LC3-II expression, as demonstrated by immunostaining analysis in aVICs (Figure 4G-I). Furthermore, the mRNA expression levels of CDKN2A and CDKN1A did not exhibit significant alterations within the 7 days following transfection, but showed a marked decrease by day 9 (Figure 4J,K). SA-GLB1 positive staining, intracellular ROS levels and SASP secretion were reduced in ATG7 OE aVICs with a markedly enhanced capability for BrdU incorporation (Figure 4L-O).

### ATG deficiency induces aVIC senescent phenotype

**Figure 5.**
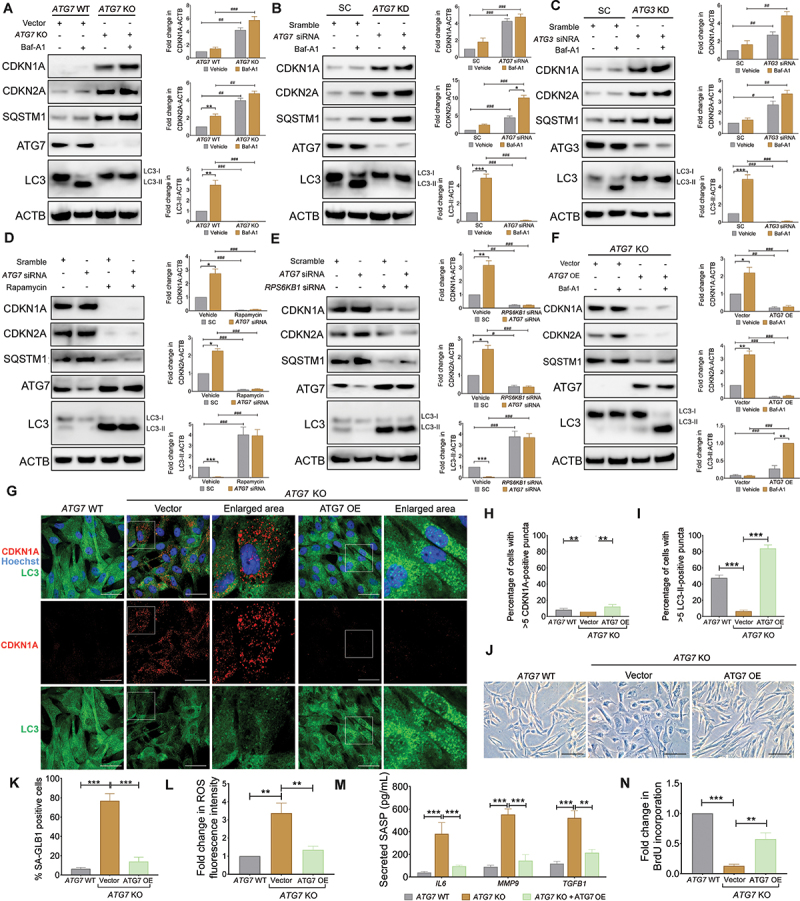
ATG deficiency induces the transformation of VIC senescent phenotype. (A-C) Representative images (left panel) of CDKN1A/p21, CDKN2A/p16, SQSTM1/p62, ATG7, ATG3, MAP1LC3/LC3 and ACTB (loading control) immunoblots of qVICs transfected with empty vectors, eSpcas9-2A-Puro (PX459)-ATG7 gRNA V2.0 plasmids (A), scramble control (SC), *ATG7*-specific siRNA (B) or *ATG3*-specific siRNA (C) in the presence or absence of 5 µm baf-A1 for 4 h (n = 3). The graphs (right panel) show the ratios of CDKN1A/p21, CDKN2A/p16, MAP1LC3/LC3-II to ACTB normalized to qVICs transfected with empty vectors in the absence of baf-A1. (D and E) Representative images (left panel) of CDKN1A/p21, CDKN2A/p16, SQSTM1/p62, ATG7, MAP1LC3/LC3 and ACTB (loading control) immunoblots of qVICs transfected with scramble or *ATG7*-specific siRNA and then treated with or without 200 nM rapamycin for 48 h (D) or *RPS6KB1*-specific siRNA for 24 h (E) (n = 3). The graphs (right panel) show the ratios of CDKN1A/p21, CDKN2A/p16, MAP1LC3/LC3-II to ACTB normalized to aVICs transfected with scramble siRNA. (F) Representative images (left panel) of CDKN1A/p21, CDKN2A/p16, SQSTM1/p62, ATG7, MAP1LC3/LC3 and ACTB immunoblots of HEK293T cells transfected with empty vectors or eSpcas9-2A-Puro (PX459)-ATG7 gRNA V2.0 plasmids and then re-expressed with or without *pcDNA3.1-Flag-ATG7* plasmids in the presence or absence of 5 µm baf-A1 for 4 h (n = 3). The graphs (right panel) show the ratios of CDKN1A/p21, CDKN2A/p16, MAP1LC3/LC3-II to ACTB. (G) Representative confocal images of CDKN1A/p21 and MAP1LC3/LC3 immunostaining in *ATG7* wild-type (WT) and *ATG7* knockout (KO) qVICs transfected with empty vectors or *pcDNA3.1-Flag-ATG7* plasmids for 24 h (n = 3). Scale bars: 50 µm. (H and I) Quantification of *ATG7* WT and *ATG7* KO qVICs (%) with > 5 CDKN1A/p21 puncta (H) or > 5 MAP1LC3/LC3-II puncta (I) transfected with empty vectors or *pcDNA3.1-Flag-ATG7* plasmids as shown in (G). (J) Representative images of SA-GLB1/β-gal staining in *ATG7* WT and *ATG7* KO qVICs transfected with empty vectors or *pcDNA3.1-Flag-ATG7* plasmids. Scale bars: 100 µm (n = 5). (K) Percentage of SA-GLB1/β-gal positive *ATG7* WT and *ATG7* KO cells transfected with empty vectors or *pcDNA3.1-Flag-ATG7* plasmids as shown in (J). (L) Quantification of ROS fluorescence intensity for *ATG7* WT and *ATG7* KO qVICs transfected with empty vectors or *pcDNA3.1-Flag-ATG7* plasmids (n = 3). (M) Quantification of secreted TGFB1, IL6 and MMP9 cytokine expression in collected supernatant from *ATG7* WT and *ATG7* KO qVIC cultures transfected with empty vectors or *pcDNA3.1-Flag-ATG7* plasmids (n = 3). (N) Quantification of BrdU incorporation for *ATG7* WT and *ATG7* KO qVICs transfected with empty vectors or *pcDNA3.1-Flag-ATG7* plasmids (n = 4). These experiments were repeated at least three times (n ≥ 3), and results are presented as mean ± SEM. ANOVA followed by Tukey’s range test. (ns, not significant; *p < 0.05; **p < 0.01; ***p < 0.001; ^#^*p* <0.05; ^##^*p* <0.01; ^###^*p* <0.001).

Autophagy impairment has been previously shown to induce cell senescence [29]. Here we demonstrate that in the presence and absence of baf-A1, *ATG7* knockout (KO) blocks autophagy flux by impairing the lipidation of MAP1LC3-I and its transformation to MAP1LC3-II in qVICs. Protein expression levels of CDKN2A, CDKN1A and SQSTM1 were found to be significantly increased with a concomitant compromise in autophagy flux by *ATG7* KO (Figure 5A). Comparable results were observed in qVICs transfected with human *ATG7*- and *ATG3*- specific siRNA (Figure 5B,C). Importantly, autophagy activation by rapamycin treatment or RPS6KB1 silencing restored autophagy flux and thereby significantly reduced CDKN2A, CDKN1A and SQSTM1 protein expression in ATG7 silenced qVICs (Figure 5D,E). In line with this finding, ATG7 re-expression in *ATG7* KO HEK293T cells restored autophagy flux and therefore reduced CDKN2A, CDKN1A and SQSTM1 protein expression (Figure 5F). These results were consistent with our immunostaining results in VICs (Figure 4G-I). ATG3 is another essential gene that facilitates the conjugation of ATG7-activated MAP1LC3 to phosphatidylethanolamine (PE) and its conversion to MAP1LC3-II [25]. Furthermore, the increased SA-GLB1 activity, intracellular ROS levels and three SASP factors (IL6, MMP9 and TGFB1) induced by *ATG7* KO were reversed in ATG7 re-expression aVICs, with a concurrent significantly increased capacity for BrdU incorporation (Figure 5J-N). Finally, the effect of autophagy inhibition on the secretome of *ATG7* KO VICs was analyzed using a cytokine array. Elevated levels of several SASP factors, including TGFB1, IL8 and TIMP2, were observed in the conditioned media of *ATG7* KO VICs compared to wild-type (WT) cells whereas certain factors, such as FGF9, CSF3/G-CSF and IGFBP4, showed reduced expression (Fig. S5). Collectively, these findings indicate that autophagy impairment drives qVICs toward a senescent phenotype.

### CDKN2A and CDKN1A localize to autophagosomes in autophagy-promoted cells

**Figure 6.**
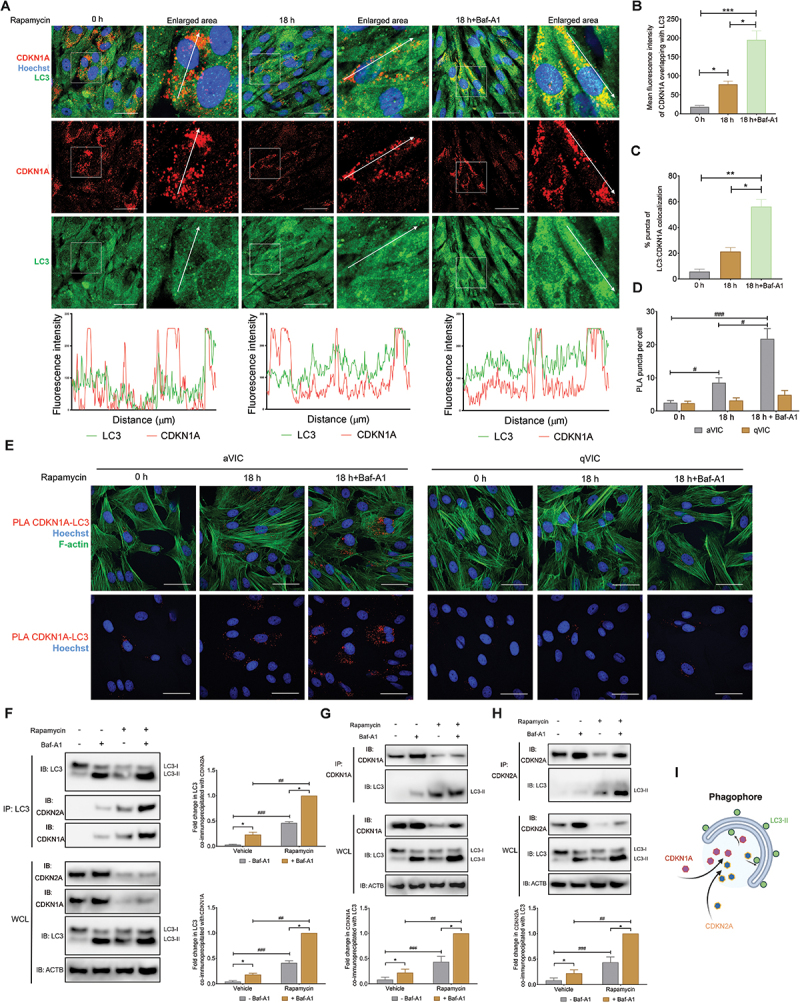
CDKN2A/p16 and CDKN1A/p21 localize on phagophores during autophagy promotion. (A) Representative confocal images (top) of CDKN1A/p21 colocalization with MAP1LC3/LC3 in aVICs treated with or without 200 nM rapamycin for 18 h. The graphs (bottom) display the fluorescence intensity in each channel over the distance (µm) depicted by the arrows. Scale bars: 50 µm. (B) Mean fluorescence of CDKN1A/p21 colocalizing with MAP1LC3/LC3 from the experiments performed in (A) (n = 6). (C) Quantification of MAP1LC3/LC3 puncta colocalizing with CDKN1A/p21 signal in cells as described in (A) (n = 6). (D) Quantification of PLA puncta per cell as described in (E). (E) Representative images of PLA for CDKN1A/p21 and the autophagosome marker MAP1LC3/LC3 in aVICs treated with or without 200 nM rapamycin for 18 h in the presence or absence of 5 µm baf-A1 for 4 h (n = 3). F-actin was stained with phalloidin. Scale bars: 50 µm. The PLA images of CDKN2A/p16 colocalization with MAP1LC3/LC3 are shown in Fig. S6. (F) Co-immunoprecipitation (Co-ip) of MAP1LC3/LC3 from aVICs treated with or without 200 nM rapamycin for 18 h in the presence or absence of 5 µm baf-A1 for 4 h. Whole-cell lysate (WCL) and immunoprecipitation complexes were analyzed by immunoblotting (left panel) with anti-CDKN1A/p21, CDKN2A/p16, MAP1LC3/LC3 and ACTB (loading control) antibodies. Quantification (right panel) of CDKN2A/p16 and CDKN1A/p21 bound to MAP1LC3/LC3, normalized to the condition in the presence of rapamycin and baf-A1, from the experiments performed in left panel. (G and H) the cells were immunoprecipitated with an anti-CDKN2A/p16 antibody (G) or anti-CDKN1A/p21 antibody (H). Immunoblots (top) for anti-CDKN1A/p21, CDKN2A/p16, MAP1LC3/LC3 and ACTB. Quantification (bottom) of MAP1LC3/LC3 bound to CDKN2A/p16 or CDKN1A/p21, normalized to the condition in the presence of rapamycin and baf-A1, from the experiments performed in left panel. (I) Schematic summarizing the binding of MAP1LC3/LC3 with CDKN1A/p21 and CDKN2A/p16. These experiments were repeated at least three times (n ≥ 3), and results are presented as mean ± SEM. ANOVA followed by Tukey’s range test. (ns, not significant; *p < 0.05; **p < 0.01; ***p < 0.001; ^#^*p* <0.05; ^##^*p* <0.01; ^###^*p* <0.001).

Promoting autophagy by ATG overexpression significantly decreased CDKN2A and CDKN1A expression. Conversely, blocking autophagy flux using ATG silencing, and knockout contributed to CDKN2A and CDKN1A accumulation. These findings suggest that both CDKN2A and CDKN1A degrade though the autophagy pathway. To further demonstrate that degradation of CDKN1A is autophagy dependent, the potential for colocalization of CDKN1A with the autophagosome marker MAP1LC3 was examined. Immunostaining studies showed endogenous CDKN2A and CDKN1A partially colocalized with MAP1LC3-II puncta in the cytoplasm of a VICs exposed to rapamycin treatment. Furthermore, this colocalization became more marked in the presence of baf-A1 (Figure 6A-C). Substances targeted for degradation via autophagy are initially encapsulated within autophagosomes, subsequently being conveyed to lysosomes where degradation ensues [25]. Proximity ligation assays (PLA) for the CDKN2A or CDKN1A and autophagosome marker MAP1LC3 further identified comparable observations in our canine cells (Figure 6D,E; Fig. S6A and S6B). Lipidation of MAP1LC3-II (membrane-bound form) from MAP1LC3-I (cytosolic form) anchors to the phagophore membrane and recognizes and sequestrates the cargoes for autophagic degradation [25]. The endogenous protein interaction between MAP1LC3 and CDKN2A or CDKN1A was therefore validated through co-immunoprecipitation (co-IP) assays. These assays demonstrated the detectability of CDKN2A and CDKN1A proteins within the immunocomplexes isolated using an anti-MAP1LC3 antibody after rapamycin exposure (Figure 6F). In an inverse co-IP assay, MAP1LC3-II was effectively co-precipitated utilizing antibodies targeting the CDKN1A and CDKN2A proteins (Figure 6G,H). These results suggest that a pool of endogenous CDKN2A and CDKN1A are localized to autophagosomes and lysosomes and sequestrated by MAP1LC3 for autophagic degradation in VICs (Figure 6I).

In addition to autophagy, cellular mechanisms for degrading biomolecules include the UPS, the primary non-lysosomal pathway for the degradation of ubiquitinated proteins. It has been established that CDKN2A and CDKN1A are also degraded via the 26S proteasome in the UPS [49,50]. To rule out the involvement of the UPS in the degradation of CDKN1A and CDKN2A, MG132, a potent inhibitor of the 26S proteasome, was employed to block proteolytic activity [51]. Our findings demonstrated that after 48 hours of MG132 treatment, the expression of PSMB5 (proteasome 20S subunit beta 5) and PSMA1 (proteasome 20S subunit alpha 1) decreased, indicating reduced proteasomal activity (Fig. S7A and S7B). Proteasomal inhibition is known to stimulate autophagy through multiple regulatory mechanisms [52–55]. Notably, the MAP1LC3-II:MAP1LC3-I ratio were increased as accompanied by the decreased CDKN1A, CDKN2A and SQSTM1, suggesting that autophagy flux increased along with reduced CDKN1A and CDKN2A (Fig. S7A-S7C). Confocal microscopy further confirmed partial colocalization of CDKN1A with MAP1LC3 following MG132 treatment (Fig. S7D), with comparable results observed for aVICs treated with the autophagy inducer torin-1 (Fig. S7D). Collectively, these data suggest that CDKN2A and CDKN1A undergo degradation via autophagy, independent of UPS involvement, in VICs.

### Autophagy receptor SQSTM1 interacts with CDKN1A and CDKN2A

Accumulating evidence suggests that the autophagic degradation of biomolecules is a distinctly selective mechanism depending on the identification of substrates by specific cargo receptors [25]. MAP1LC3 plays a key role in selective autophagy by interacting with cargo receptors which have MAP1LC3-interacting region (LIR) to bind with MAP1LC3 and facilitate the sequestration of cargo substrates into the phagophore [25]. To exclude the possibility that CDKN1A and CDKN2A are sequestered within autophagosomes through direct interaction with MAP1LC3-II through their LIR, the presence of an LIR motif in either canine or human CDKN2A and CDKN1A proteins was investigated using an online database (https://ilir.warwick.ac.uk) [56]. No LIR sequences were identified in CDKN2A and CDKN1A in either species, suggesting the degradation of CDKN2A and CDKN1A may occur through selective autophagy.

**Figure 7.**
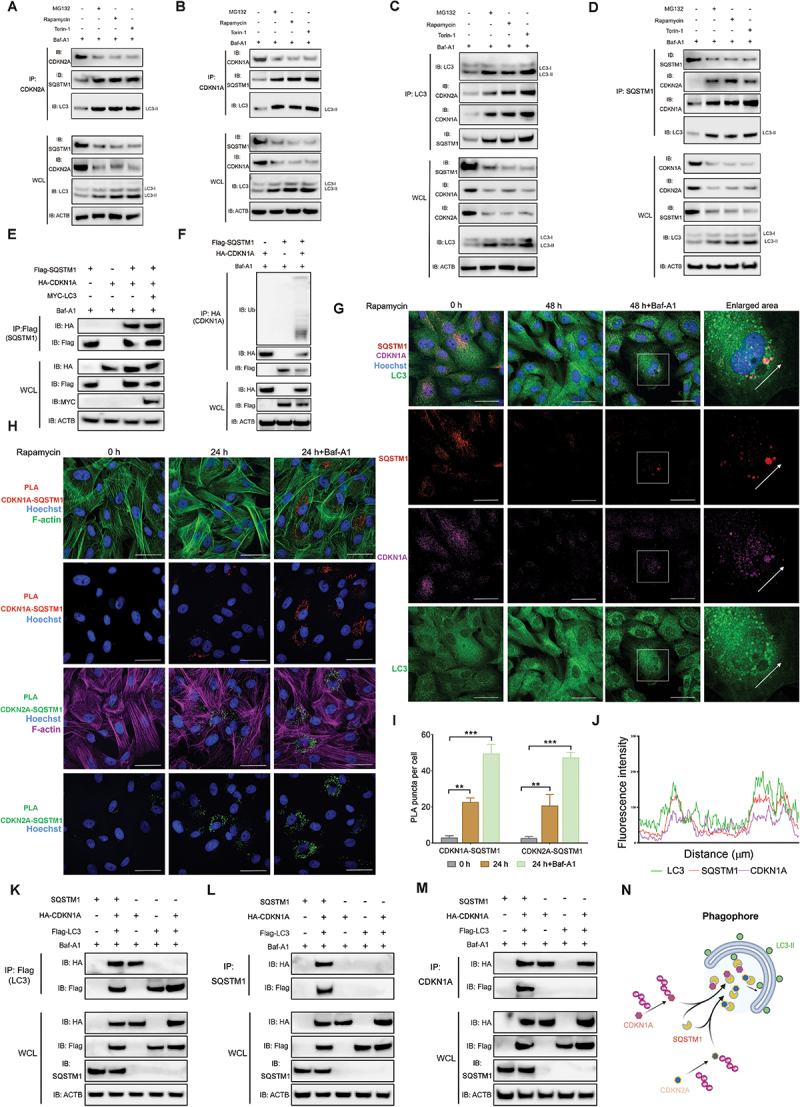
CDKN2A/p16 and CDKN1A/p21 interacts with autophagy receptor SQSTM1/p62. (A and B) Co-immunoprecipitation of CDKN1A/p21 and CDKN2A/p16 from aVICs treated with 10 µm MG132 for 36 h, 200 nM rapamycin for 24 h, or 80 nM torin-1 for 18 h following 5 µm baf-A1 treatment for 4 h. Whole-cell lysate (WCL) and immunoprecipitation complexes were analyzed by immunoblotting with anti-CDKN1A/p21, CDKN2A/p16, SQSTM1/p62, MAP1LC3/LC3 and ACTB (loading control) antibodies (n = 2). (C and D) the cells were immunoprecipitated with an anti-MAP1LC3/LC3 antibody and anti-SQSTM1/p62 antibody. WCL and immunoprecipitation complexes were analyzed by immunoblotting with anti-CDKN1A/p21, CDKN2A/p16, SQSTM1/p62, MAP1LC3/LC3 and ACTB (n = 2). (E) HEK293T cells were co-transfected with *pcDNA3.1-HA-CDKN1A/p21*, *pcDNA3.1-MYC-MAP1LC3/LC3* and *pcDNA3.1-Flag-SQSTM1/p62* for 24 h in the presence of 5 µm baf-A1 for 4 h. Flag, HA, MYC and ACTB (loading control) immunoblots for IP of Flag from HEK-293T cells and the corresponding WCL (n = 3). (F) IP of HA from aVIC co-transfected with *pcDNA3.1-HA-CDKN1A/p21* and *pcDNA3.1-Flag-SQSTM1/p62* in the presence of 5 µm baf-A1 for 4 h. Immunoblots for anti-ha, Flag, ubiquitin (Ub), and ACTB (n = 3). (G) Representative confocal images of CDKN1A/p21 colocalization with SQSTM1/p62 and MAP1LC3/LC3 in aVICs treated with or without 200 nM rapamycin for 48 h in the absence or presence of 5 µm baf-A1 for 4 h (n = 3). (H) PLA for CDKN1A/p21, CDKN2A/p16 and SQSTM1/p62 on aVICs treated with or without 200 nM rapamycin for 18 h in the presence of 5 µm baf-A1 for 4 h (n = 3). F-actin was stained with phalloidin. Scale bars: 50 µm. (I) Number of PLA dots (CDKN1A/p21-SQSTM1/p62 and CDKN2A/p16-SQSTM1/p62) per cell. (J) the graphs display the fluorescence intensity in each channel (as shown in G) over the distance (µm) depicted by the arrows. (K-M) HEK293T cells were co-transfected with *pcDNA3.1-SQSTM1/p62*, *pcDNA3.1-HA-CDKN1A/p21* and *pcDNA3.1-Flag-MAP1LC3/LC3*. Flag, HA, SQSTM1/p62 and ACTB (loading control) immunoblots for the three-way IP of SQSTM1/p62, MAP1LC3/LC3, CDKN1A/p21 from HEK-293T cells in the presence of baf-A1 and the corresponding whole-cell lysate (WCL) (n = 2). (N) Schematic summarizing the recruitment of cargoes CDKN2A/p16 and CDKN1A/p21 to phagophores by SQSTM1/p62 sequestration. These experiments were repeated at least two times (n ≥ 2), and results are presented as mean ± SEM. ANOVA followed by Tukey’s range test. (ns, not significant; *p < 0.05; **p < 0.01; ***p < 0.001; ^#^*p* <0.05; ^##^*p* <0.01; ^###^*p* <0.001).

Elevated protein levels of the autophagy receptor SQSTM1, along with its accumulation within immature autophagosomes, were detected in senescent aVICs exhibiting increased levels of CDKN2A and CDKN1A. This indicates that SQSTM1 May mediate the selective degradation of CDKN2A and CDKN1A. To examine the protein interactions between MAP1LC3, SQSTM1, CDKN2A and CDKN1A, aVICs were treated with rapamycin, MG132 and torin-1, followed by co-IP assays. Baf-A1 was used to abolish the degradation of cargo-receptor-MAP1LC3-II complex in autolysosomes. Our results demonstrated that endogenous SQSTM1 interacted with both MAP1LC3 and CDKN2A or CDKN1A in aVICs (Figure 7A-D). Additionally, HEK-293T cells were co-transfected with *pcDNA3.1-MAP1LC3-MYC*, *pcDNA3.1-CDKN2A-HA* (or *pcDNA3.1-CDKN1A-HA*), and a Flag-tagged plasmid construct encoding SQSTM1. Co-IP assays in HEK-293T cells further confirmed that exogenous CDKN2A, CDKN1A and MAP1LC3-II were detected in the immunocomplexes isolated with anti-SQSTM1 antibody in HEK293T cells (Figure 7E; Fig. S8A). The mechanism of SQSTM1-mediated selective autophagy is highly conserved across eukaryotic species, from yeast to mammals. SQSTM1 contains several conserved domains, including LIR and ubiquitin-associated (UBA) domain, to exert its conserved functions in selective autophagy [25]. SQSTM1 recognizes and binds to polyubiquitin chains tagged on cargoes for autophagic degradation by its UBA structural domain [57]. The ubiquitination of CDKN2A and CDKN1A and their interaction with SQSTM1 were examined by co-IP assays. Polyubiquitin linkages on CDKN2A and CDKN1A can be only detected in immunocomplexes isolated from cells co-transfected with SQSTM1 and CDKN2A or CDKN1A, indicating that the ubiquitination CDKN2A and CDKN1A cargoes interact with SQSTM1 (Figure 7F; Fig. S8B). Furthermore, it was demonstrated that SQSTM1 colocalized with MAP1LC3-II and CDKN1A puncta in the cytoplasm of aVICs (Figure 7G,J). Additionally, PLA for SQSTM1 and either CDKN2A or CDKN1A confirmed the colocalization of these proteins (Figure 7H,I). This interaction was further confirmed by co-IP assays isolating the exogenously over-expressed cargo-receptor complex with anti-Flag (MAP1LC3), anti-SQSTM1 and anti-HA (CDKN2A or CDKN1A) antibodies in HEK 293T cells (Figure 7K-M; Fig. S8C-S8E). Taken together, these results suggest that CDKN2A and CDKN1A are sequestrated by SQSTM1 in autolysosomes for autophagic degradation (Figure 7N).

### Autophagy receptor SQSTM1 mediates CDKN1A and CDKN2A degradation

**Figure 8.**
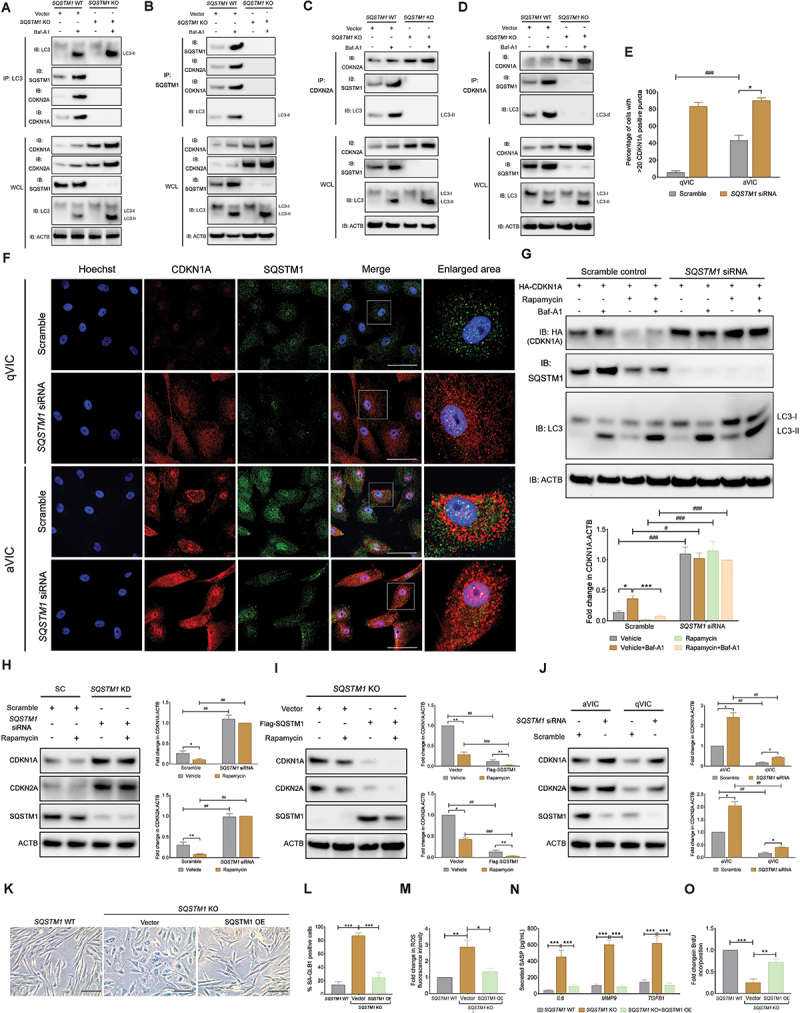
The degradation of CDKN1A/p21 and CDKN2A/p16 is mediated by autophagy receptor SQSTM1/p62. (A-D) Co-ip of MAP1LC3/LC3, SQSTM1/p62, CDKN2A/p16 and CDKN1A/p21 from *SQSTM1/p62* WT and *SQSTM1/p62* KO aVICs in the presence or absence of 5 µm baf-A1 for 6 h. Whole-cell lysate (WCL) and immunoprecipitation complexes were analyzed by immunoblotting with anti-CDKN1A/p21, CDKN2A/p16, SQSTM1/p62, MAP1LC3/LC3 and ACTB (loading control) antibodies (n = 3). (E) Quantification of aVICs and qVICs (%) with > 20 CDKN1A/p21 puncta transfected with scramble or SQSTM1/p62-specific siRNA as shown in (F). (F) Representative confocal images of CDKN1A/p21 and SQSTM1/p62 in aVICs and qVICs transfected with scramble or *SQSTM1/p62*-specific siRNA for 24 h (n = 3). (G) Representative images (top) of HA (CDKN1A/p21), SQSTM1/p62, MAP1LC3/LC3 and ACTB (loading control) immunoblots of HEK293T cells transfected with empty vectors or *pcDNA3.1-HA-CDKN1A/p21* plasmids in the presence or absence of 200 nM rapamycin for 48 h and/or 5 µm baf-A1 for 4 h (n = 3). CDKN1A/p21:ACTB ratio (bottom) normalized to SQSTM1/p62 knockdown (KD) HEK293T treated with rapamycin and baf-A1. (H) Representative images (left panel) of CDKN1A/p21, CDKN2A/p16, SQSTM1/p62 and ACTB (loading control) immunoblots of aVICs transfected with scramble or *SQSTM1/p62*-specific siRNA in the presence or absence of 200 nM rapamycin for 48 h (n = 6). CDKN1A/p21, CDKN2A/p16:ACTB ratio (right panel) normalized to SQSTM1/p62 knockdown (KD) aVICs treated with rapamycin. (I) Representative images (left panel) of CDKN1A/p21, CDKN2A/p16, SQSTM1/p62 and ACTB (loading control) immunoblots of *SQSTM1/p62* KO HEK293T cells transfected with empty vector or *pcDNA3.1-Flag-SQSTM1/p62* plasmids in the presence or absence of 200 nM rapamycin for 48 h (n = 3). CDKN1A/p21, CDKN2A/p16:ACTB ratio (right panel) normalized to *SQSTM1/p62* KO HEK293T cells in the absence of rapamycin. (J) Representative images (left panel) of CDKN1A/p21, CDKN2A/p16, SQSTM1/p62 and ACTB (loading control) immunoblots of aVICs and qVICs transfected with scramble or *SQSTM1/p62*-specific siRNA 24 h (n = 6). CDKN1A/p21, CDKN2A/p16:ACTB ratio (right panel) normalized to aVICs with scrambles. (K) Representative images of SA-GLB1/β-gal staining in *SQSTM1/p62* WT and *SQSTM1/p62* KO qVICs transfected with empty vectors or *pcDNA3.1-Flag-SQSTM1/p62* plasmids. Scale bars: 100 µm (n = 3). (L) Percentage of SA-GLB1/β-gal positive cells. (M) Quantification of ROS fluorescence intensity for *SQSTM1/p62* WT and *SQSTM1/p62* KO qVICs transfected with empty vectors or *pcDNA3.1-Flag-SQSTM1/p62* plasmids (n = 3). (N) Quantification of secreted TGFB1, IL6 and MMP9 cytokine expression detected by ELISA in collected supernatant from qVIC cultures transfected with empty vectors or *pcDNA3.1-Flag-SQSTM1/p62* plasmids (n = 3). (O) Quantification of BrdU incorporation for *SQSTM1/p62* WT and *SQSTM1/p62* KO qVICs transfected with empty vectors or *pcDNA3.1-Flag-SQSTM1/p62* plasmids (n = 4). These experiments were repeated at least three times (n ≥ 3), and results are presented as mean ± SEM. ANOVA followed by Tukey’s range test. (ns, not significant; *p < 0.05; **p < 0.01; ***p < 0.001; ^#^*p* <0.05; ^##^*p* <0.01; ^###^*p* <0.001).

To further investigate if the autophagic degradation of CDKN2A and CDKN1A proteins is controlled by the SQSTM1-mediated autophagic process the expression of SQSTM1 was ablated in canine aVICs. Co-IP assays showed that SQSTM1 deficiency abolished the physical binding of CDKN2A or CDKN1A cargoes with MAP1LC3-II (autophagosomes) in the presence or absence of baf-A1 (Figure 8A-D). Consistent with these findings, SQSTM1 knockdown resulted in a marked upregulation of CDKN1A levels in both aVICs and qVICs (Figure 8E,F). This observation was further confirmed in HEK293T cells co-transfected with *SQSTM1* siRNA and *pcDNA3.1-CDKN1A-HA* (Figure 8G). Rapamycin induced exogenous CDKN2A and CDKN1A degradation was only detectable in HEK-293T cells treated with scramble control siRNA, but not in SQSTM1-silenced cells (Figure 8G), suggesting that CDKN2A and CDKN1A fail to be sequestrated by SQSTM1 for autophagic degradation. Similarly, the accumulation of endogenous CDKN2A and CDKN1A was observed in SQSTM1 silenced qVICs and HEK293T cells (Figure 8H), whereas SQSTM1 re-expression in SQSTM1 silenced HEK293T cells restored CDKN2A and CDKN1A degradation following rapamycin treatment (Figure 8I). Immunostaining for SQSTM1 showed that the knockdown of SQSTM1 expression increased CDKN2A and CDKN1A accumulation in aVICs, and that this effect became more marked in non-senescent qVICs (Figure 8J), consistent with our immunoblotting data (Figure 8E,F). Furthermore, re-expressing SQSTM1 in SQSTM1-deficient qVICs reversed the increased SA-GLB1 activity, intracellular ROS levels and SASP secretion, and concurrently increased the cells’ capacity for BrdU incorporation (Figure 8K-O). Collectively, these results suggest that CDKN2A and CDKN1A are degraded by SQSTM1-meidated selective autophagy.

## Discussion

In this study we have identified that deficient autophagy flux and autophagosome maturation are important characteristics of senescent aVICs harvested from dogs clinically affected by myxomatous mitral valve degeneration, suggesting that these processes may underpin the pathogenesis of this disorder. MTOR antagonism decreased the expression of two main cyclin-dependent kinase inhibitors CDKN2A and CDKN1A and attenuated cell senescence. Autophagy promotion by overexpressing ATG genes alleviated cell senescence, whereas induction of autophagy deficiency caused senescence-associated phenotype with high expression of SASP. Importantly, we report a novel degradation pathway for cyclin-dependent kinase inhibitors CDKN2A and CDKN1A, mediated by the autophagy-lysosomal pathway in VICs. Furthermore, CDKN2A and CDKN1A can be ubiquitinated and then sequestrated by the autophagy receptor SQTSM1 and eventually degraded by selective autophagy.

Interestingly, short-term rapamycin treatment resulted in decreased nuclear levels of CDKN2A and CDKN1A, accompanied by their accumulation in the cytoplasm. Prolonged exposure to rapamycin, however, led to an overall reduction in their expression, suggesting that rapamycin may facilitate the translocation of CDKN2A and CDKN1A from the nucleus to the cytoplasm. Nonetheless, real-time confirmation of this translocation requires time-lapse confocal microscopy to monitor the dynamic movement of exogenously tagged eGFP- or mCherry-CDKN2A and CDKN1A in live cells. While the precise mechanism of rapamycin-induced translocation remains unclear, previous studies have shown that phosphorylation of specific residues in CDKN1A, such as Thr145 by activated PI3K-AKT, reduces its affinity for nuclear import and enhances its retention in the cytoplasm [58]. This interaction sequesters CDKN1A in the cytoplasm, preventing its reentry into the nucleus. Our findings suggest that rapamycin-mediated inhibition of RPS6KB1 activates AKT through a negative feedback loop, and this increased AKT activity may explain the promotion of CDKN1A cytoplasmic localization. Although the mechanism of CDKN2A’s subcellular localization is not as well-characterized as CDKN1A, the previous study suggests that post-translational modifications may regulate its intracellular distribution [59]. Moreover, in the present study, CDKN1A and CDKN2A displayed puncta-like structures in autophagy-deficient VICs as observed in confocal microscopy images. Previous research has shown that impaired autophagy leads to the abnormal accumulation of proteins normally degraded through autophagic pathways. These proteins may form intracellular aggregates, which exhibit higher molecular weight and larger volume compared to their monomeric forms [60]. Defective synucleinphagy has been implicated in the impaired degradation of SNCA/α-synuclein, resulting in its aggregation in Parkinson disease [26]. Further experiments are needed to determine the composition of these aggregates, specifically to differentiate between the monomeric and polymeric forms of CDKN2A and CDKN1A.

Our findings demonstrated that the MTOR inhibitor rapamycin induced cell proliferation in aVICs, accompanied by a simultaneous reduction in SASP. While rapamycin has been previously shown to reduce SASP without reversing the senescence-induced growth arrest in human fibroblasts [61,62], in certain contexts – particularly in cells with activation of the PI3K-AKT-MTOR signaling pathway, such as certain cancer cells and aVICs – selective inhibition of MTOR-RPS6KB1 signaling can trigger AKT activation through a negative feedback loop from RPS6KB1 to IRS1-PI3K-AKT. Activated AKT promotes cell growth, cell proliferation and survival. Cancer cells can exploit this mechanism to counteract the anti-proliferative effects of MTOR-dependent anticancer drugs, including rapamycin and its analogs CCI-779 and RAD001 [24,63]. Furthermore, studies demonstrated that SASP expression is affected by cell senescent state or directly regulated by MTOR signaling [64]. To eliminate the influence of rapamycin-induced cell proliferation on SASP, we employed torin-1, a highly potent and selective ATP-competitive MTOR inhibitor that directly targets both MTORC1 and MTORC2. Torin-1 inhibits cell growth and proliferation to a significantly greater extent than the MTORC1-specific inhibitor rapamycin [42], and was used in the present study to effectively suppress cell proliferation. Torin-1 treatment demonstrated a potent anti-proliferative effect and showed a comparable effect with rapamycin on SASP expression. Moreover, rapamycin induced cell proliferation was abolished with the addition of torin-1, with a concomitant decreased SASP expression. Our data therefore suggest that the reduction in SASP expression results from MTOR inhibition.

Impaired autophagy has been known as an important hallmark of cell aging with autophagic activity typically declining with age [30]. This decline contributes to the accumulation of damaged cellular components which can contribute to cellular dysfunction [65]. The reduced autophagy in older cells is believed to be a significant factor in the aging process and age-related diseases [25]. Our recent study found that aVICs exhibit cell senescence with impaired autophagy in myxomatous mitral valve tissues, as evidenced by increased CDKN1A but reduced ATG7 expression. The senescent aVICs showed significantly increased protein expression of cyclin-dependent kinase inhibitor CDKN1A, CDKN2A, TP53 and SASP, while ATG7 and MAP1LC3-II expression were significantly decreased [4]. In the present study we extend these findings to show that senescent aVICs exhibit compromised autophagy flux and immature autophagosomes indicating deficient autophagy activity in MMVD. The autophagic deficiency and senescent VIC transition have been previously reported to be initiated by the activation of MTOR-RPS6KB1 signaling induced by TGFBs, which importantly are also the primary drivers of MMVD pathogenesis [24]. Senescent cells commonly show increased activity of SA-GLB1, which is an enzyme located in lysosomes [66]. Increased GLB1 activity reflects the altered lysosomal acidic environment and function as it is detected at sub-optimal pH (pH 6) and which is higher than the optimal acidic pH for lysosomal enzymes [67]. GLB1 activity also indicates increased lysosomal content, including size and number of lysosomes [66]. This is in agreement with our observations on lysosomal expansion in MMVD derived aVICs.

Autophagy plays a crucial role in maintaining cellular health. Indeed, the protective role of autophagy has been identified in many age-related degenerative disorders, particularly neurodegenerative diseases [25]. In a transgenic mouse model of Parkinson disease, microglia facilitate the degradation of neuron-secreted human SNCA/α-synuclein via autophagic pathways. Simultaneously, autophagy governs the decomposition of amyloid-beta (Aβ) fibrils and modulates Aβ-evoked inflammasome activities *in vitro* within microglial cultures [26,68]. In a related valvulopathy to MMVD, induction of autophagy improves the elimination of dead cells and ameliorates the calcified phenotype in calcific aortic valve stenosis (CAVS) [69]. Here, we have shown promoting autophagy by ATG3 and ATG7 overexpression significantly clears excessive cellular CDKN2A and CDKN1A and attenuates cell senescence in aVICs. Important to MMVD, autophagy is the key contributing factor for senescent cells resistance to apoptosis and aberrant ECM remodeling [64]

Cell senescence can act as a response to impaired autophagy, especially in the context of aging and age-related diseases [29]. Chronic blockage of autophagic flux has been shown to induce cellular senescence in murine fibroblasts [30], and is also adequate to provoke early onset senescence via a ROS- and TP53-dependent pathway in primary human fibroblasts [29]. Defective autophagy also accelerates senescence in vascular smooth muscle cells [30]. Inhibition of microglial autophagy leads to reduced cell proliferation and increased CDKN1A and SASP expression in a mouse model of Alzheimer disease [28]. Furthermore, disruption of autophagy in aged satellite cells, or genetically induced autophagy deficiency in young cells, leads to cellular senescence while restoration of autophagic activity reverses the senescent state and reinstates regenerative capabilities in aged satellite cells [70]. Consistent with these observations, in our study selective knockdown or knockout of *ATG3* and *ATG7* compromises autophagy and results in accumulation of CDKN2A and CDKN1A in aVICs and HEK293T cells, whereas rapamycin, RPS6KB1 silencing and ATG7 re-expression restore autophagy flux and reduce CDKN2A, CDKN1A, SASP expression and decrease SA-GLB1 activity. Although the aberrant accumulation in cellular components caused by impaired autophagy is considered as the trigger for cell senescence in naturally aging systems, the underlying mechanism remains unknown and warrants future exploration. It is now widely acknowledged that senescence represents a heterogeneous cellular state, influenced by the cell type of origin and the nature of the stress inducing it. Given the association of autophagy with the regulation of senescence, metabolism and secretion, it is plausible to hypothesize that the specific form of senescence exhibits distinct characteristics with unique effects on the local microenvironment [29,71]. In certain contexts, such as during oncogene-induced senescence (OIS), autophagy is upregulated and collaborates with the TOR-autophagy spatial coupling compartment (TASCC) to support the synthesis of secretory proteins [71].

One important characteristic of cell senescence is irreversible proliferation arrest, largely mediated by activation of either one or both of the TP53-CDKN1A and CDKN2A-RB1 pathways [64]. CDKN2A and CDKN1A are the key downstream functional transcription factors negatively regulating cell cycle progression as CDKIs [64]. CDKN1A has been shown to be degraded through the UPS [72]. CDKN1A can be recognized and ubiquitinated by the SCF (Skp1-Cullin-F-box) complex and degraded by 26S proteasomes [73]. This study is the first to report that CDKN1A and CDKN2A localize to autophagosomes for autophagic degradation. Importantly, this may explain how proliferating qVICs with a high level of autophagy flux can sustain basal levels of CDKN1A a CDKN2A expression without inducing cell-cycle arrest.

In recent years the understanding of autophagy has expanded beyond merely encompassing macroautophagy in which the cargo recognition for autophagic degradation lacks specificity [74]. Autophagy is currently acknowledged for its heightened specificity in determining the selection of cargo for degradation [75]. This variant of selective autophagy may hold greater significance in safeguarding against numerous mammalian diseases compared to macroautophagy, which predominantly transpires in response to nutritional deficiency. Unlike macroautophagy the recognition of cellular cargoes for degradation in selective autophagy requires the participation of specific autophagy receptors [76]. In the present study we have identified SQSTM1 as the specific autophagy receptor for CDKN2A and CDKN1A degradation. The silencing of SQSTM1 inhibits the autophagic degradation of CDKN2A and CDKN1A and increases their accumulation suggesting the autophagic degradation of CDKN2A and CDKN1A mediated by SQSTM1. More recently to reflect selectivity, distinct varieties of selective autophagy have been commonly identified by prefixes that originate from their respective cargo, such as mitophagy (mitochondria), ER-phagy (endoplasmic reticulum), and lipophagy (lipids) [25]. Considering the pivotal role of CDKN2A and CDKN1A in the initiation and perpetuation of cellular senescence, and their selective autophagic degradation mediated by SQSTM1, we would suggest the term “senophagy” to describe this novel autophagy-related phenomenon. This term would align with the term “senolytic” to denote a category of specialized anti-senescence pharmaceuticals designed to target and eliminate senescent cells [77]. However, the question of whether other cyclin-dependent kinase inhibitors such as CDKN1B/p27^KIP1^ and TP53 undergo degradation through a comparable pathway requires further investigation.

The current study has clarified the interplay between autophagy and senescence and has identified the selective autophagy pathway for CDKN2A and CDKN1A degradation in both canine mitral VICs and HEK293T cells. Considering autophagy is a highly conserved cellular process sharing core autophagy-lysosomal machinery and regulatory *ATG* genes across a broad spectrum of organisms [74], we would speculate that this mechanism is active in additional cell types and species, for example in human valvulopathies and other age-related degenerative disorders. Since valve interstitial cells are a specialized type of fibroblast [78–81], it would be worthwhile to investigate if SQSTM1-mediated CDKN2A and CDKN1A selective autophagy has fibroblast specificity, not least since senescent transformation contributes to many the pathological changes in many age-related diseases, such as kidney fibrosis, atherosclerosis and skin aging [80,82,83].

**Figure 9.**
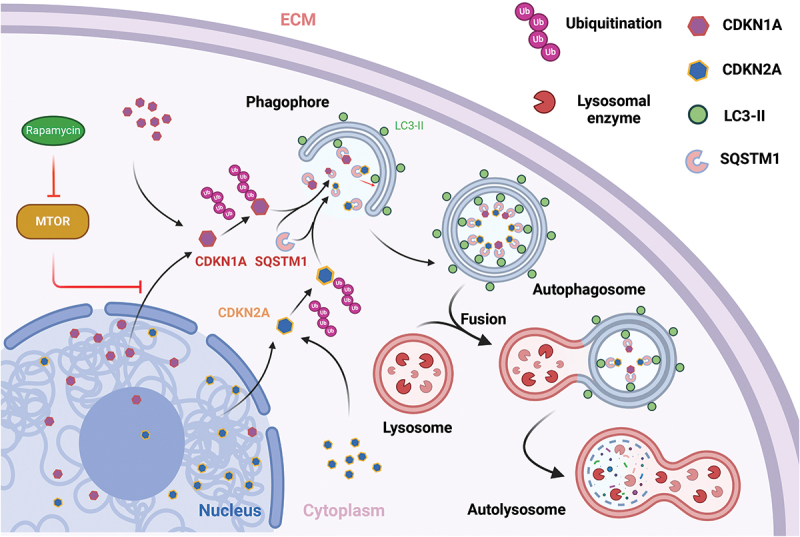
Schematic illustration of the proposed model for the autophagic degradation of CDKN1A/p21 and CDKN2A/p16.

In conclusion, this study demonstrates that autophagy regulates the transformation of the VIC senescent phenotype. Notably, autophagy facilitates the relocation of both CDKN2A and CDKN1A from the nucleus to the cytoplasm, where they undergo degradation through a SQSTM1-mediated selective autophagic pathway (as illustrated in Figure 9). These findings suggest that focusing on senescence and autophagy in VICs represents a feasible therapeutic strategy for myxomatous mitral valve degeneration in both dogs and humans.

## Materials and methods

### Clinical samples and ethics statement

Mitral valves were obtained from six dogs of various breeds, all diagnosed with MMVD, and six healthy young adult dogs of varied breeds, at the Hospital for Small Animals, The Royal (Dick) School of Veterinary Studies, University of Edinburgh. The excised valves were categorized in terms of their gross pathological characteristics, utilizing the Whitney classification system, which ranges from grade 0 (normal) to 4 (disease). This evaluation was independently conducted by two observers [84]. For the purpose of this investigation, all six MMVD-afflicted canines were categorized as Whitney grade 2, indicative of moderate disease severity.

All clinical sample collections were executed in adherence to the protocols established by the Veterinary Ethics Research Committee (Institutional Care and Use Committee; project No. 96/21). Prior to collection, written consent was obtained from each dog owner, ensuring compliance with ethical norms. None of the dogs were euthanized expressly for the objectives of this study. written consent was secured from each dog owner, ensuring adherence to ethical standards.

### Primary VIC isolation and culture

VICs were collected from entire valves of dogs diagnosed with grade 2 mmVD, indicative of moderate affliction, paralleling the isolation of healthy VICs from valves of healthy dogs. The process entailed rapid excision and dissection of canine mitral valve leaflets, followed by phenotyping and preparation for cell culture as described in our previous study [24].

### Cell line

HEK293T (ATCC, CRL-11268) cells were maintained in DMEM medium (Gibco 11,995,065) supplemented with 10% fetal bovine serum (Gibco, A5256801) and 1% penicillin-streptomycin (Gibco 15,140,122).

### Antibodies and reagents

Rabbit anti-ATG7 (10088–2-AP), anti-SQSTM1/p62 (18420–1-AP), anti-HA (51064–2-AP), anti-Flag (20543–1-AP), anti-MYC (16286–1-AP), anti-CDKN2A/p16 (10883–1-AP), anti-CDKN1A/p21(10355–1-AP), anti-TUBB/β-tubulin (10094–1-AP), anti-histone-H3 (17168–1-AP), anti-ubiquitin (10201–2-AP) antibodies were purchased from Proteintech. Rabbit anti-ATG5 (12994), anti-LAMP1 (9091), anti-ACTB/β-actin (4967) were purchased from Cell Signaling Technology. Rabbit anti-ATG3 (ab108251) were purchased from Abcam. Rabbit anti-MAP1LC3 (PM036) antibodies were purchased from MBL. Mouse anti-SQSTM1/p62 (66184–1-Ig), anti-CDKN1A/p21 (67362–1-Ig), anti-LAMP1 (67300–1-Ig) and -LAMP2 (66301–1-Ig) antibodies were purchased from Proteintech. Mouse anti-CDKN2A/p16 (ab54210) antibodies were purchased from Abcam. Rat anti-CDKN2A/p16 (ab241543) and anti-CDKN1A/p21 (ab107099) antibodies were purchased from Abcam. Secondary antibodies conjugated to horseradish peroxidase (HRP) were purchased from Proteintech (SA00001–2; SA00001–1). Phalloidin-iFluor 488 Reagent (ab176753) was purchased from Abcam.

Rapamycin (13346), torin-1 (10997), MG132 (10012628) and bafilomycin A_1_ (11038) were obtained from Cayman Chemical. Dimethylsulfoxide (DMSO; 276855) was purchased from Sigma Aldrich. TGFB1 (10804-HNAC) was purchased from Sino Biological.

### Constructs

Human *ATG3*, *ATG7*, *SQSTM1* and *RPS6KB1* cDNA were cloned into the pcDNA3.1+ vector (Addgene 208,051; deposited by Quanfu Ma) with the Flag tag, intended for use in gene overexpression assays. cDNAs for human and canine CDKN2A/p16 and CDKN1A/p21 were cloned into the pcDNA3.1-HA vector (Addgene 128,034; deposited by Oskar Laur). The human *MAP1LC3* cDNA was also cloned, either with a MYC or Flag tag, into the pcDNA3.1 vector. The *SQSTM1* cDNA was inserted into the pcDNA3.1 vector without any tagging. To achieve gene knockout of *ATG7*, and *SQSTM1*, the eSpCas9-2A-Puro (PX459) V2.0 plasmids, sourced from Genscript (designed by Feng Zhang’s lab), were utilized. Finally, the integrity and accuracy of all these constructed plasmids were confirmed through DNA sequencing.

### Gene overexpression

Cells (1 × 10^6^) were plated in six-well plates per well and incubated overnight. A total of 10 µg of plasmids containing the cDNA sequences for human *ATG3*, *ATG7*, *RPS6KB1*, and canine *CDKN2A* and *CDKN1A* (Genscript) were introduced to cells using the Neon transfection system (Invitrogen, NEON1S). Cells transfected with empty pcDNA3.1 vectors or subjected to transfection without vectors were used as a vector and mock control. The gene expression was validated (Fig. S9A-S9F). In this study, human *ATG3*, *ATG7*, and *RPS6KB1* cDNA were used due to the high degree of homology shared between canine and human *ATG3*, *ATG7*, and *RPS6KB1* genes.

### RNA interference

Cells (1.0 × 10^6^) were transfection with a total amount of 1 µM human *RPS6KB1* siRNA (Santa Cruz Biotechnology, sc -36,165), human *ATG7* siRNA (Santa Cruz Biotechnology, sc -41,447), human *ATG3* siRNA (Santa Cruz Biotechnology, sc -72,582), mouse SQTSM1 (Santa Cruz Biotechnology, sc -29,828), or scrambled control siRNA (Santa Cruz Biotechnology, sc -37,007) using Lipofectamine 3000 (Invitrogen, L3000001) transfection reagents, following the manufacturer’s guidelines. Cells transfected with scrambled siRNA served as a scramble control. The knockdown efficiency was validated (Fig. S9G-S9J). The *ATG3*, *ATG7*, and *RPS6KB1* genes exhibit high evolutionary conservation across mouse, human, and dog, thereby justifying the use of these siRNAs in this study.

### CRISPR-Cas9 knockout

Single guide RNA (sgRNA) was designed for *ATG7* and *SQSTM1* gene target sequence and then cloned into the eSpCas9-2A-Puro (PX459) V2.0 plasmids (Genscript; designed by Feng Zhang’s lab) using seamless cloning technology. HEK293T cells and dogs VICs were transfected with the constructs using Neon electroporation system (Invitrogen, NEON1S). At 36 h after transfection, DNA was extracted from a fraction of polyclonal cells and then subjected to PCR amplification for gene target sequences. PCR products were exposed to T7 Endonuclease I (T7EI; New England Biolabs, M0302) and agarose gel electrophoresis to validate gene knockout (Fig. S9K-S9N). The other portion of cells were exposed to puromycin (10 μg/ml) for 72 h monoclonal selection. Immunoblotting assays were performed for the identification of monoclonal cells. The sgRNA and primers utilized for PCR amplification are recorded in **Table S1**.

### Immunofluorescence assay

Immunofluorescence assays were performed as described previously [24]. Briefly, cells adhered to glass coverslips underwent fixation and subsequent permeabilization, followed by a blocking with 5% normal goat serum at room temperature (RT). Post-blocking, primary antibodies were applied to cells, followed by incubation at 4°C overnight. Cells were then incubated with Alexa Fluor 488 anti-rabbit (1:500; Invitrogen, A-11034), Alexa Fluor 647 anti-mouse (1:500; Invitrogen, A-31571) and/or Alexa Fluor 555 anti-rat (1:300; Invitrogen, A-21434) antibody. Subsequently, Hoechst (1:1000; Sigma 62,249) were used for nuclear visualization. Confocal images were captured using a confocal microscope (Zeiss LSM 710) and processed by ImageJ. Concurrently, negative controls were processed by replacing the primary antibody with control rabbit IgG (Abcam, ab172730) at equivalent concentrations. This control step was implemented to ascertain the specificity of the primary antibody binding.

### Live cell imaging

Cells were seeded onto glass coverslips. After 200 nM rapamycin treatment for 72 h, culture medium was replaced with DMEM supplemented with the LysoBrite Deep Red probe (AAT Bioquest 22,646). Cells were incubated at 37°C for 30 min. The loading medium was replaced with the fresh medium and live cell imaging was performed using a high-resoluton confocal microscope (Zeiss LSM 880). Captured images were processed by ImageJ.

### Immunoblotting assay

Immunoblotting assays were performed as described previously [24]. Cells were collected and lysed in RIPA lysis buffer (Thermo Scientific 89,900), followed by the quantification of protein concentration using rapid BCA protein assay (Thermo Scientific, A55860). Protein lysates were separated by gel electrophoresis and translocated onto nitrocellulose membranes (Thermo Scientific 88,018). The membranes underwent a blocking phase with 5% (w:v) bovine serum albumin (BSA; Thermo Scientific, J10857–18) in phosphate-buffered saline (Thermo Scientific 10,010,049) with 0.1% Tween-20 (Sigma Aldrich, P1379; PBST) for 1 h. This was followed by an overnight incubation at 4°C with primary antibodies. After the primary antibody incubation, membranes were exposed to HRP-conjugated secondary antibodies at RT for 1 h. The development of these membranes was carried out using the GeneGenome system (Syngene) and quantification was performed using ImageJ.

### Co-ip assay

Harvested cells were lysed using IP lysis buffer (Thermo Scientific 87,787). This was followed by sonication of the cell lysates for a brief duration of 10 s. The lysates were then subjected to centrifugation at 12,000 g for 25 min, post which the supernatants were carefully collected. The protein content within these supernatants was determined quantitatively utilizing BCA reagents (Thermo Scientific, A55860). A specified aliquot of the supernatant was allocated for utilization in whole cell extract assays. For immunoprecipitation, 500 µg of the lysates were incubated with 3 µg of the designated antibodies at 4°C overnight. This was followed by an additional incubation with 12 µl of protein A/G sepharose beads (Abcam, ab206996) at 4°C for 4 h. Subsequently, the beads were subjected to three washing steps using the IP lysis buffer. After washing, the beads were resuspended in 10 µl of 4×SDS-PAGE loading buffer, then heated at 95°C for 8 min. Finally, the samples were processed for immunoblotting analysis.

### BrdU assay

Cell proliferation was assessed by analyzing the capability of BrdU incorporation. BrdU assays were performed as previously described [24]. Cells were plated and incubated with BrdU for 3 h. Following BrdU incorporation, cells were fixed, permeated and then subject to DNA denaturation. Subsequently, the cells were incubated with an anti-BrdU antibody (Abcam, ab126556), followed by the incubation with a HRP-conjugated secondary antibody. Finally, the development of a colorimetric reaction indicative of cell proliferation was quantitatively measured at 450 nm.

### Cellular ROS assay

The level of cellular ROS was evaluated using the DCFDA/H2DCFDA cellular ROS assay kit (Abcam, ab113851) according to the manufacturer’s protocol. Cells were grown at a density of 3 × 10^4^ in a dark, clear bottom 96-well microplate. Cells were washed and incubated with DCFDA solution for 45 min at 37°C in the dark. DCFDA solution was then removed and replaced with culture medium. Microplate was measured immediately on a fluorescence plate reader (BioTek Cytation 3, Agilent).

### Cellular senescence assay

The assay (Sigma Aldrich, KAA002) for SA-GLB1/β-gal activity was conducted in line with the manufacturer’s guidelines. Cells were plated and fixed by 0.25% (v:v) glutaraldehyde and then subjected to staining. Images were captured and processed by ImageJ.

### PLA

Colocalization analysis was further confirmed by PLA assays (Sigma Aldrich, DUO92101) according to the manufacturer’s protocol. Briefly, cells were cultured on coverslips and then subject to fixation and permeabilization. The fixed cells were then exposed to Duolink blocking buffer, followed by incubation with various combinations of primary antibodies. The coverslips were then incubated with anti-rabbit PLUS (Sigma Aldrich, DUO92002) and anti-mouse MINUS (Sigma Aldrich, DUO92004) secondary antibodies connected with PLA probes. The PLA detection reagent (Sigma Aldrich, DUO9008) was used for PLA signal detection. The coverslips were then stained with phalloidin (Abcam, ab176753), and DNA was visualized using Hoechst 33,342 (Sigma Aldrich 62,249). Imaging of the samples was performed using an inverted ZEISS 880 high-resolution confocal microscope. The quantification of PLA dots was carried out through analysis with ImageJ software.

### Quantitative real-time PCR (qRT-pcr)

qRT-PCR were conducted as previously described previously [24]. The primers for these assays were provided by Integrated DNA Technologies (IDT) and their sequences were listed in **Table S2**.

### Enzyme-linked immunosorbent assay (ELISA)

The cell culture medium was collected, diluted and then subjected to ELISA assays. The detection for TGFB1 (Invitrogen, BMS249–4), MMP9 (Invitrogen, BMS2016–2) and IL6 (Invitrogen, EH2IL6) were performed according to the manufacturer’s guidelines.

### Nuclear and cytosolic fractionation assay

Cells were processed according to the nuclear and cytoplasmic extraction kit (Thermo Scientific 78,833), followed by immunoblotting analysis.

### STEM

VICs were subjected to treatment with vehicle DMSO, rapamycin or torin-1, followed by fixation in 3% glutaraldehyde prepared in 0.1 M sodium cacodylate buffer. Sample preparation and sectioning for STEM analysis were conducted according to previously established protocols [85]. Specimens mounted on nickel mesh grids were examined using a Zeiss Gemini 360 scanning electron microscope, and electron micrographs were obtained with an annular STEM detector.

### Autophagosome maturation detection

The pcDNA3.1±mCherry-eGFP-MAP1LC3B plasmid was synthesized by Genscript. After 3 days post electroporation, VICs were treated with vehicle DMSO, rapamycin or torin-1 for 18 h. Cells were fixed with 4% paraformaldehyde for 30 min at room temperature and nuclei were counterstained with Hoechst 33,342 (Sigma 62,249). Images were captured with a ZEISS LSM880 high-resolution confocal microscopy.

### Cytokine arrays

Conditioned media from WT and *ATG7* KO qVICs were collected and analyzed using the C5 cytokine antibody array (RayBiotech, AAH-CYT-5) for the semi-quantitative detection of 80 proteins, following the manufacturer’s protocol and as previously described [86]. Signal intensities were quantified, normalized to internal positive controls, and expressed as log2-fold changes, where fold change represents the relative expression of *ATG7* KO compared to WT qVICs. The heatmap was generated using an online bioinformatics tool [87], employing bidirectional hierarchical clustering with the complete linkage method to determine cluster distance based on the furthest pair of data points within clusters. Euclidean distance was used as the metric to assess similarity between data points. The heatmap function, implemented with a callback function, visualized the clustered data through color gradients, representing expression patterns across both rows and columns, with clustering applied to enhance interpretability of the relationships between data points. A comprehensive list of the cytokines included is provided in Table S3.

### Statistical analysis

Statistical analysis was conducted by one-way analysis of variance (ANOVA) followed by Tukey’s range test using GraphPad Prism 8. *p* value of less than 0.05 was considered statistically significant.

## Supplementary Material

Supplementary file Revised 20250214 R5.docx

## Data Availability

Data will be available upon reasonable requests.
