# Supplementary material for: Autophagy regulates cellular senescence by mediating the degradation of CDKN1A/p21 and CDKN2A/p16 through SQSTM1/p62-mediated selective autophagy in myxomatous mitral valve degeneration
Source: Autophagy. 2025 Mar 4;21(7):1433–55. doi: 10.1080/15548627.2025.2469315 (PMC12283023; doi:10.1080/15548627.2025.2469315)
Supplement: Supplementary file Revised 20250214 R5.docx [file KAUP_A_2469315_SM4296.docx]

**Table S1**. The sgRNAs and primers used for *ATG7* and *SQSTM1* knockout in VICs and HEK293T cells.

|  | *ATG7* | *SQSTM1* |
| --- | --- | --- |
| sgRNA target sequence (5ʹ-3ʹ) | AGAAGAAGCTGAACGAGTAT | AGGGCTTCTCGCACAGCCGC |
| Forward sequence  (5ʹ-3ʹ) | GTTGTGTTTCAAGGTAGCCTG | GGGCTTGCACCGGGGGCACACC |
| Reverse sequence  (5ʹ-3ʹ) | CACTGATTATAAAAGACAAGG | GGGCACCAGGAAGGTGGGGGG |

**Table S2**. The primers used for qRT-PCR.

| Gene | Forward primer (5ʹ-3ʹ) | | | Reverse primer (5ʹ-3ʹ) | | |
| --- | --- | --- | --- | --- | --- | --- |
| *GAPDH* | | ATCACTGCCACCCAGAAGAC | | | TCAGCTCAGGGATGACCTTG | |
| *IL6* | | TTAAGTACATCCTCGGCAAAATCT | | | CAGTGCCTCTTTGCTGTCTTCA | |
| *IL1B* | | CAAGTCTCCCACCAGCTCTGTA | | | GGGCTTCTTCAGCTTCTCCAA | |
| *TNF/TNF-α* | | TCTCGAACCCCAAGTGACAAG | | | CAACCCATCTGACGGCACTA | |
| *MMP9* | | TGAGAACTAATCTCACTGACAAGCA | | | GCTCGGCCACTTGAGTGTA | |
| *TGFB1* | | CAAGGATCTGGGCTGGAAGTGGA | | | CCAGGACCTTGCTGTACTGCGTGT | |
| *TGFB2* | | GCAGCAAGACGATAATCACG | | | TCTTGTCGCTGTCGTCCTC | |
| *TGFB3* | | CTGGCCCTTTACAACAGCAC | | | CGACTCGGTGTTTTCCTGAG | |
| *CDKN2A/p16* | | GGTCGGAGCCCGATTCA | | | ACGGGGTCGGCACAGTT | |
| *CDKN1A/p21* | | | ACCTCTCAGGGCCGAAAAC | | | TAGGGCTTCCTCTTGGAGAA |

**Table S3**. List of cytokines and abbreviations.

| **#** | **Approved name** | **Approved symbol** |
| --- | --- | --- |
| **1** | angiogenin | ANG |
| **2** | brain derived neurotrophic factor | BDNF |
| **3** | C-X-C motif chemokine ligand 13 | CXCL13/BLC |
| **4** | C-C motif chemokine ligand 23 | CCL23 |
| **5** | epidermal growth factor | EGF |
| **6** | C-X-C motif chemokine ligand 5 | CXCL5/ENA-78 |
| **7** | C-C motif chemokine ligand 11 | CCL11/Eotaxin-1 |
| **8** | C-C motif chemokine ligand 24 | CCL24/Eotaxin-2 |
| **9** | C-C motif chemokine ligand 26 | CCL26/Eotaxin-3 |
| **10** | fibroblast growth factor 4 | FGF4 |
| **11** | fibroblast growth factor 6 | FGF6 |
| **12** | fibroblast growth factor 7 | FGF7 |
| **13** | fibroblast growth factor 9 | FGF9 |
| **14** | fms related receptor tyrosine kinase 3 | FLT3 |
| **15** | C-X3-C motif chemokine ligand 1 | CX3CL1 |
| **16** | C-X-C motif chemokine ligand 6 | CXCL6/GCP2 |
| **17** | colony stimulating factor 3 | CSF3/GCSF |
| **18** | glial cell derived neurotrophic factor | GDNF |
| **19** | colony stimulating factor 2 | CSF2/GM-CSF |
| **20** | C-X-C motif chemokine ligand 1 | CXCL1/GROa |
| **21** | C-X-C motif chemokine ligand 2 | CXCL2/GROb |
| **22**  **23** | C-X-C motif chemokine ligand 3  hepatocyte growth factor | CXCL3/GROg  HGF |
| **24** | C-C motif chemokine ligand 1 | CCL1 |
| **25** | interferon gamma | IFNG |
| **26** | insulin like growth factor 1 | IGF1 |
| **27** | insulin like growth factor binding protein 1 | IGFBP1 |
| **28** | insulin like growth factor binding protein 2 | IGFBP2 |
| **29** | insulin like growth factor binding protein 3 | IGFBP3 |
| **30** | insulin like growth factor binding protein 4 | IGFBP4 |
| **31** | interleukin 1 alpha | IL1A |
| **32** | interleukin 1 beta | IL1B |
| **33** | interleukin 2 | IL2 |
| **34** | interleukin 3 | IL3 |
| **35** | interleukin 4 | IL4 |
| **36** | interleukin 5 | IL5 |
| **37** | interleukin 6 | IL6 |
| **38** | interleukin 7 | IL7 |
| **39** | interleukin 8 | IL8 |
| **40** | interleukin 10 | IL10 |
| **41** | interleukin 12 | IL12 |
| **42** | interleukin 13 | IL13 |
| **43** | interleukin 15 | IL15 |
| **44** | interleukin 16 | IL16 |
| **45** | C-X-C motif chemokine ligand 10 | CXCL10/IP10 |
| **46** | leptin | LEP |
| **47** | LIF interleukin 6 family cytokine | LIF |
| **48** | TNF superfamily member 14 | TNFSF14 |
| **49** | C-C motif chemokine ligand 2 | CCL2/MCP1 |
| **50** | C-C motif chemokine ligand 8 | CCL8/MCP2 |
| **51** | C-C motif chemokine ligand 7 | CCL7/MCP3 |
| **52** | C-C motif chemokine ligand 13 | CCL13/MCP4 |
| **53** | colony stimulating factor 1 | CSF1/M-CSF |
| **54** | C-C motif chemokine ligand 22 | CCL22/MDC |
| **55** | macrophage migration inhibitory factor | MIF |
| **56** | C-X-C motif chemokine ligand 9 | CXCL9/MIG |
| **57** | C-C motif chemokine ligand 4 | CCL4/MIP1β |
| **58** | C-C motif chemokine ligand 15 | CCL15/MIP1δ |
| **59** | C-C motif chemokine ligand 20 | CCL20/MIP3α |
| **60** | pro-platelet basic protein | PPBP/NAP2 |
| **61** | neurotrophin 3 | NTF3/NT3 |
| **62** | neurotrophin 4 | NTF4/NT4 |
| **63** | oncostatin M | OSM |
| **64** | secreted phosphoprotein 1 | SPP1/OPN |
| **65** | TNF receptor superfamily member 11b | TNFRSF11B/OPG |
| **66** | C-C motif chemokine ligand 18 | CCL18/PARC |
| **67** | platelet derived growth factor subunit B | PDGFB |
| **68** | placental growth factor | PGF/PLGF |
| **69** | C-C motif chemokine ligand 5 | CCL5/RANTES |
| **70** | KIT ligand | KITLG/SCF |
| **71** | C-X-C motif chemokine ligand 12 | CXCL12/SDF1α |
| **72** | C-C motif chemokine ligand 17 | CCL17/TARC |
| **73** | transforming growth factor beta 1 | TGFB1 |
| **74** | transforming growth factor beta 2 | TGFB2 |
| **75** | transforming growth factor beta 3 | TGFB3 |
| **76** | thrombopoietin | THPO |
| **77** | TIMP metallopeptidase inhibitor 1 | TIMP1 |
| **78** | TIMP metallopeptidase inhibitor 2 | TIMP2 |
| **79** | tumor necrosis factor | TNF/TNFα |
| **80** | lymphotoxin alpha | LTA/TNFβ |
| **81** | vascular endothelial growth factor A | VEGFA |


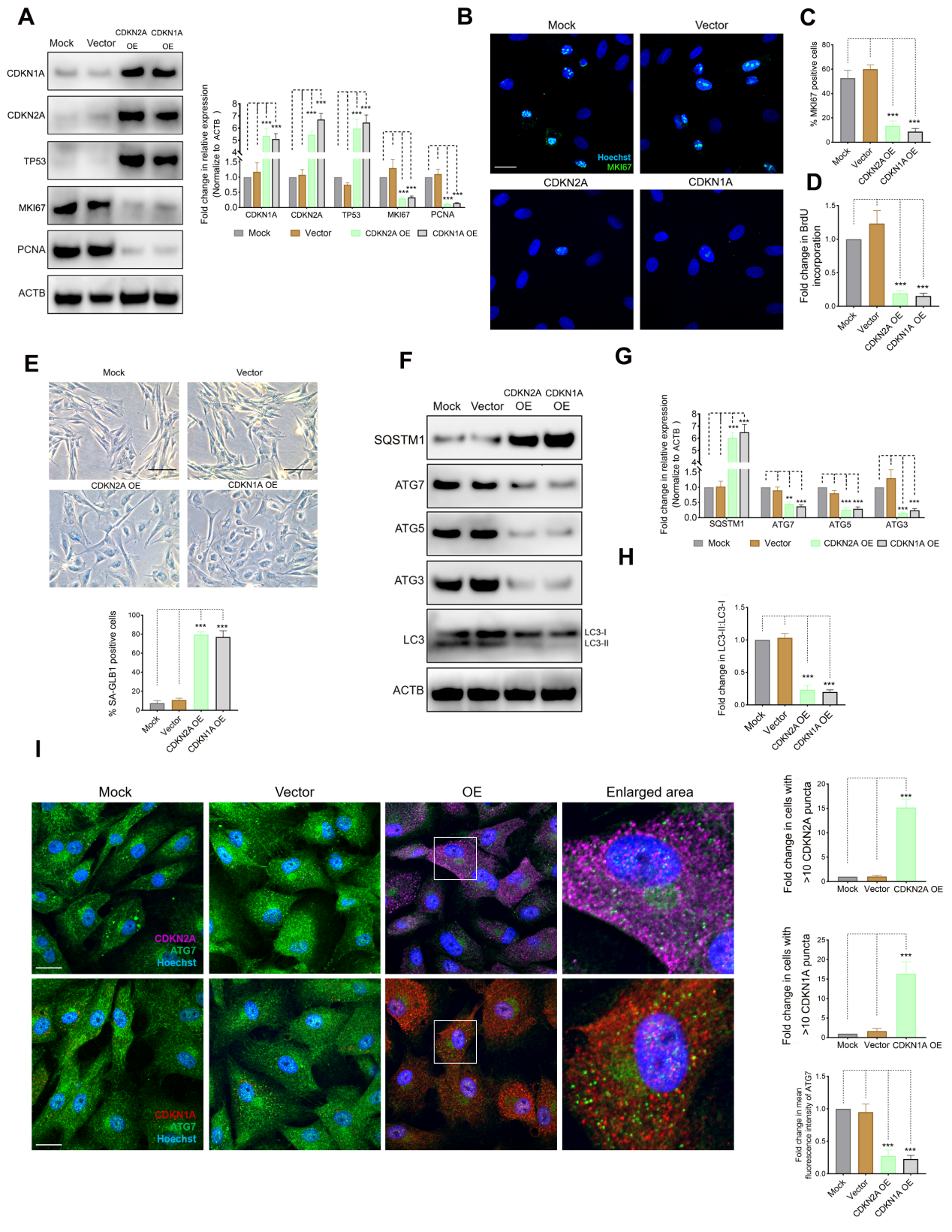


**Figure S1.** Overexpression of CDKN1A/p21 and CDKN2A/p16 induces the senescent phenotype of VICs with compromised autophagy. (**A**) Representative images (left panel) of anti-CDKN1A, -CDKN2A, -TP53/p53, -MKI67/Ki-67, -PCNA and -ACTB (loading control) immunoblots of qVICs transfected with empty vectors (vector), canine *pcDNA3.1-Flag-CDKN2A* (CDKN2A OE), canine *pcDNA3.1-Flag-CDKN1A* (CDKN1A OE), or subjected to transfection without vectors (mock). The graphs (right panel) show the ratios of CDKN1A, CDKN2A, TP53, MKI67, PCNA to ACTB normalized to the mock (n=3). (**B**) Representative images of MKI67 immunostaining in mock, vector, CDKN2A OE or CDKN1A OE. Scale bars: 50 µm (n=3). (**C**) Percentage of MKI67-positive cells as shown in (**B**). (**D**) Quantitation of BrdU incorporation in mock, vector, CDKN2A OE or CDKN1A OE (n=4). (**E**) Representative images (top) and quantification (bottom) of SA-GLB1/β-gal positive cells in mock, vector, CDKN2A OE or CDKN1A OE. Scale bars: 100 µm (n=6). (**F**) Immunoblots for anti-SQSTM1, ATG3, ATG5, ATG7, MAP1LC3/LC3 and ACTB of mock, vector, CDKN2A OE and CDKN1A OE. (**G**) Quantification of the tested proteins: ACTB, normalized to the mock (n=3). (**H**) Ratio of MAP1LC3/LC3-II to MAP1LC3/LC3-I in mock, vector, CDKN2A OE and CDKN1A OE. (**I**) Confocal imaging of cells immunostained with ATG7, CDKN1A or CDKN2A, (left panel) and quantification (right panel) of ATG7, CDKN1A or CDKN2A in mock, vector, CDKN2A OE or CDKN1A OE. Scale bars: 50 µm (n=3). Results are presented as mean ± SEM. ANOVA followed by Tukey's range test. (*p < 0.05; **p < 0.01; ***p < 0.001).


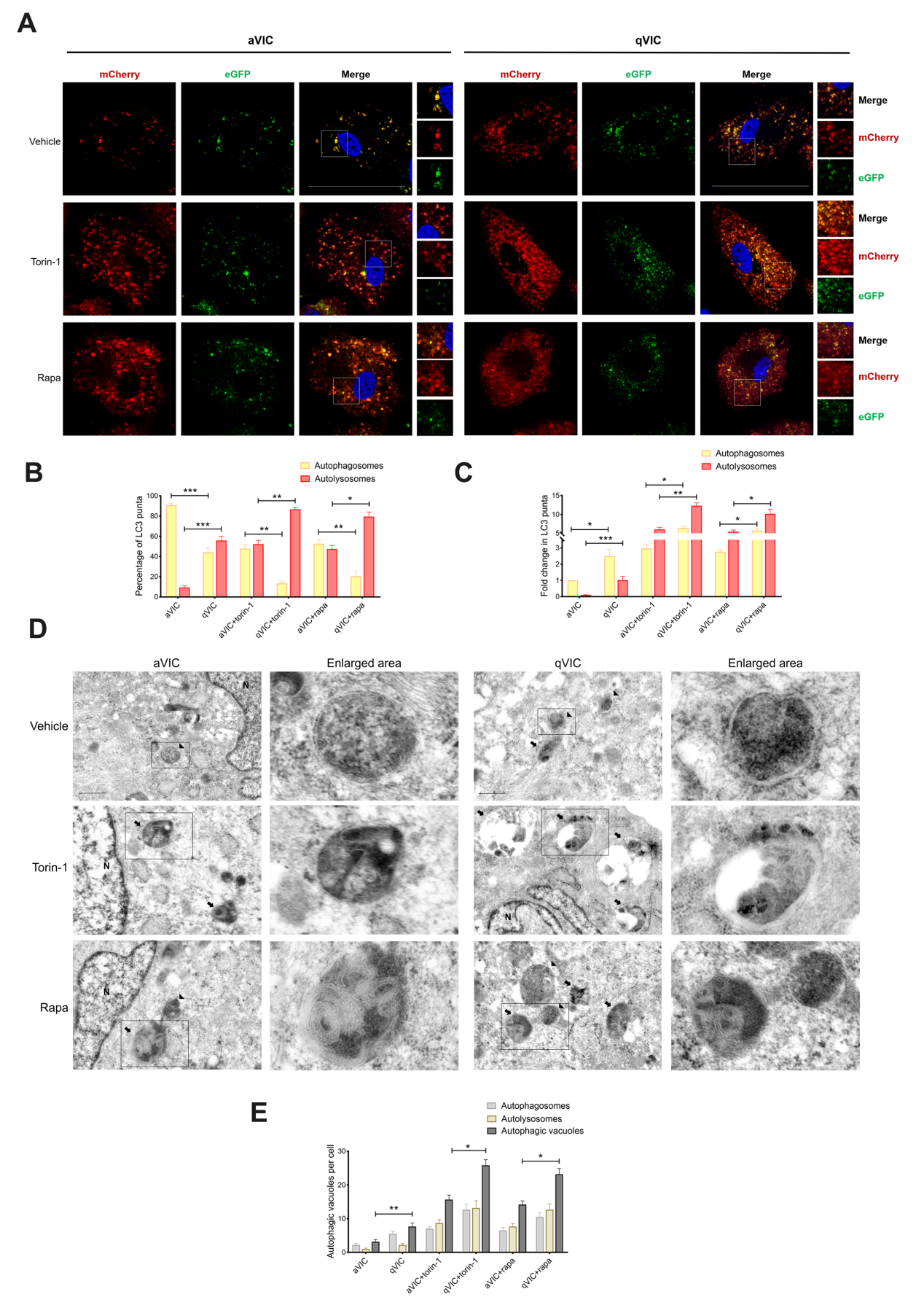


**Figure S2**. Autophagosome maturation is evaluated using the mCherry-eGFP-MAP1LC3B/LC3B reporter system and STEM in VICs. (**A**) Representative images of VICs expressing the mCherry-eGFP-MAP1LC3B/LC3B construct were obtained under both control conditions and following treatment with 80 nM torin-1 or 200 nM rapamycin (rapa) for 18 h (n=3). Insets display enlarged views of the regions marked by white boxes. Scale bars: 50 µm. (**B**) Quantification of the proportion of MAP1LC3/LC3 puncta corresponding to autophagosomes (yellow) and autolysosomes (red) in the cells. (**C**) Quantification of the total number of MAP1LC3/LC3 puncta representing autophagosomes (yellow) and autolysosomes (red) in the cells. (**D**) Representative STEM images of autophagosomes and autolysosomes in VICs following treatment with 80 nM torin-1 or 200 nM rapamycin (rapa) for 18 h. Black arrowheads indicate double-membrane autophagosomes and black arrows mark autolysosomes. The number of autophagic vacuoles (autophagosomes and autolysosomes) per cell was calculated. N, nucleus. Scale bar: 100 nm (n=3). (**E**) Quantification of the total number of autophagosomes, autolysosomes and autophagic vacuoles in the cells. Results are presented as mean ± SEM. ANOVA followed by Tukey's range test. (*p < 0.05; **p < 0.01; ***p < 0.001).


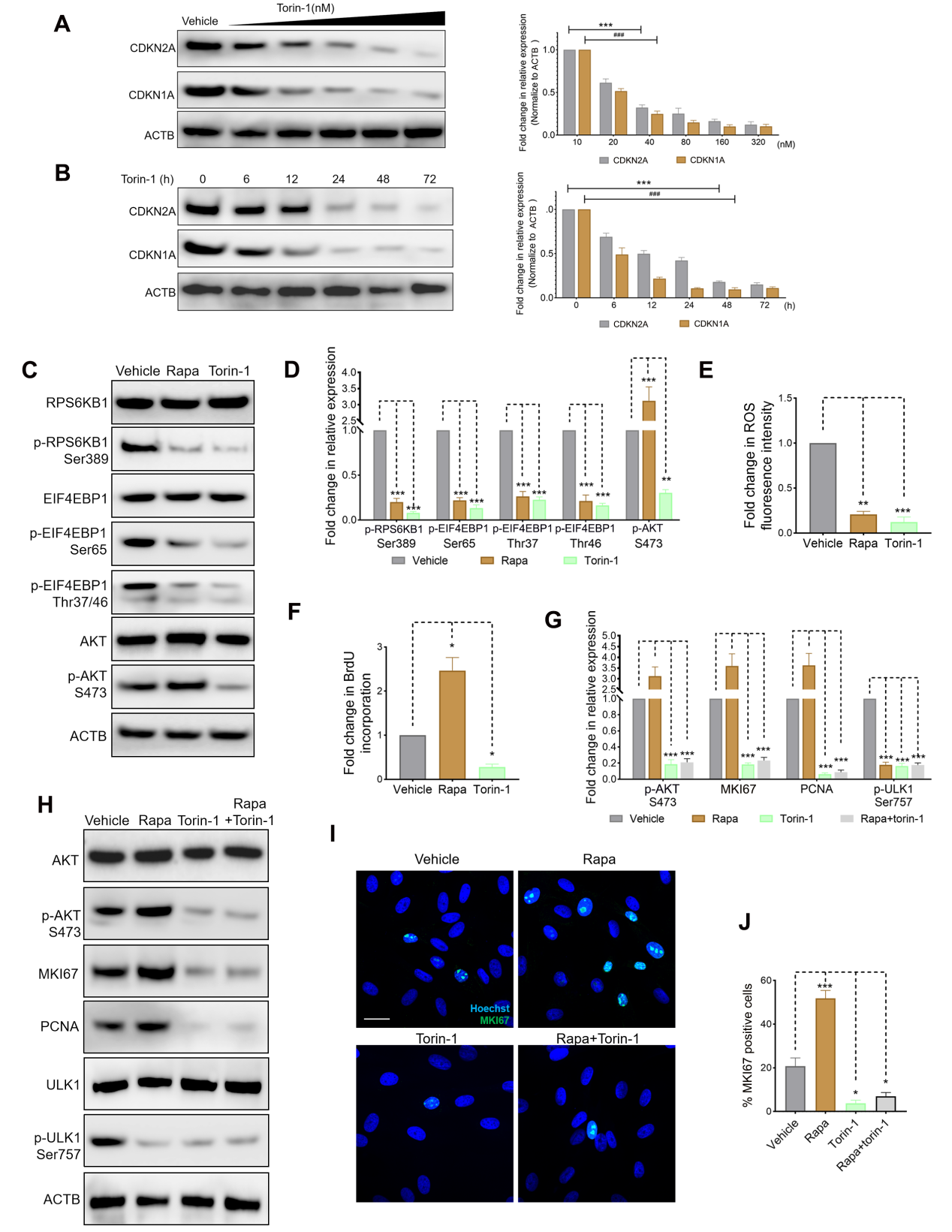


**Figure S3.** The effect of rapamycin and torin-1 on MTOR activity and cell senescence. (**A**) Representative images (left panel) of CDKN1A/p21, CDKN2A/p16 and ACTB (loading control) immunoblots of aVICs treated with the indicated dose ranges of torin-1 (10, 20, 40, 80, 160 or 320 nM) for 48 h (n=3). CDKN1A and CDKN2A:ACTB ratio (right panel) in cells, normalized to aVICs treated with vehicle ethanol. (**B**) Representative images (left panel) of CDKN1A, CDKN2A and ACTB immunoblots of aVICs treated with 80 nM torin-1 for the indicated time periods (n=3). CDKN1A and CDKN2A:ACTB ratio (right panel) in cells, normalized to aVICs treated with torin-1 at 0 h. (**C**) Immunoblots (left panel) for anti-RPS6KB1, p-RPS6KB1, EIF4EBP1, p-EIF4EBP1, AKT, p-AKT and ACTB of aVICs treated with 200 nM rapamycin (rapa) or 80 nM torin-1 for 48 h. (**D**) Quantification of the phosphorylated:total proteins normalized to aVICs treated with vehicle. (**E**) Quantification of ROS fluorescence intensity for aVICs treated either with rapamycin or torin-1 (n=4). (**F**) Quantification of BrdU incorporation for aVICs treated either with rapamycin or torin-1 (n=4). (**G**) Quantification of the tested proteins:ACTB, or phosphorylated:total proteins, normalized to aVICs treated with vehicle, and the respective immunoblots are shown in (**H**). (**H**) Representative images of anti-MKI67/Ki-67, PCNA, ULK1, p-ULK1, AKT, p-AKT and ACTB immunoblots of aVICs treated with rapamycin, torin-1 or a combination of both. (**I**) Representative images of MKI67 immunostaining in aVICs treated with rapamycin, torin-1 or a combination of both. Scale bars: 50 µm (n=6). (**J**) Percentage of MKI67-positive cells as shown in (**I**). Results are presented as mean ± SEM. ANOVA followed by Tukey's range test. (*p < 0.05; **p < 0.01; ***p < 0.001).


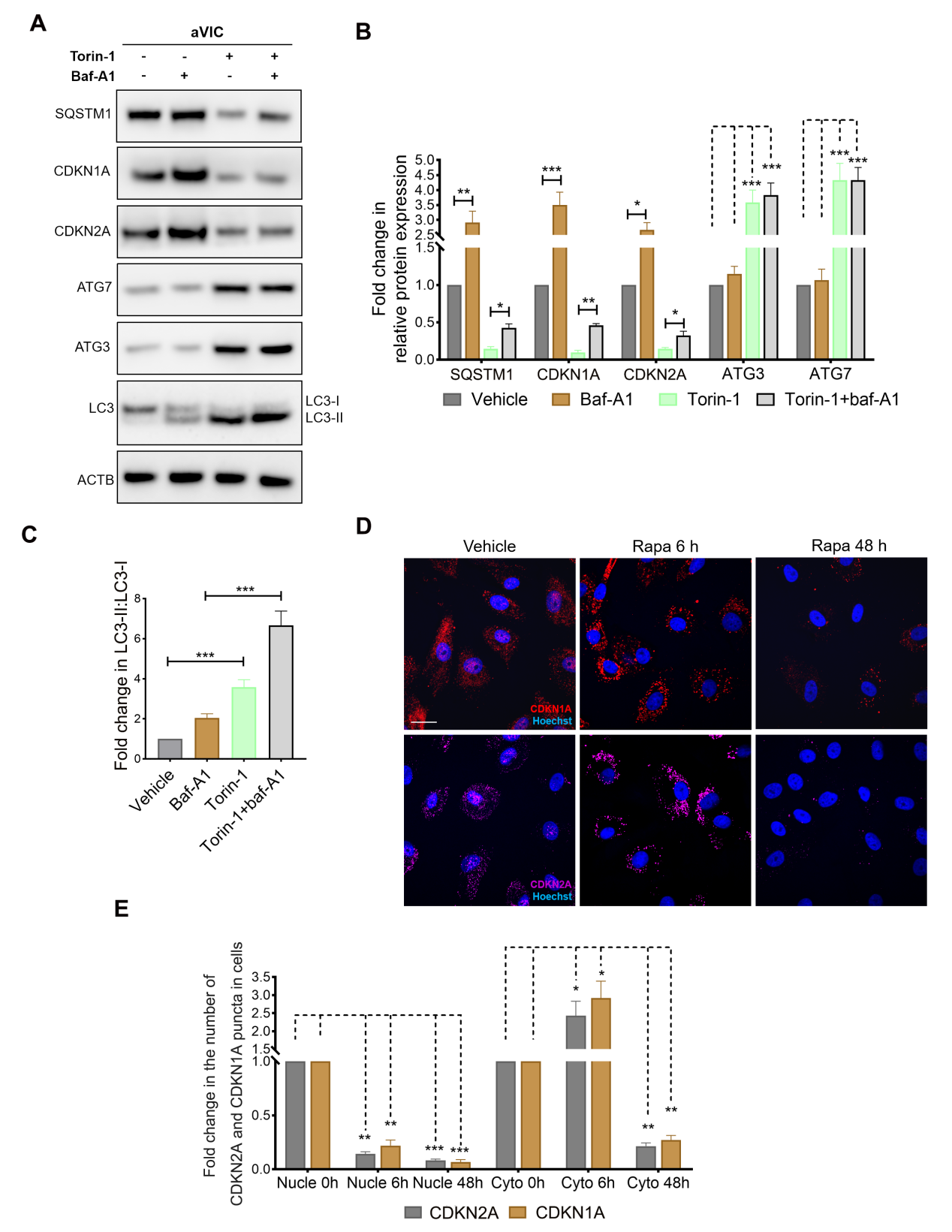


**Figure S4.** Torin-1 promotes autophagy flux and the effect of rapamycin on the expression of CDKN2A/p16 and CDKN1A/p21 in nucleus and cytoplasm. (**A**) Representative images of anti-SQSTM1/p62, CDKN1A, CDKN2A, ATG7, ATG3, MAP1LC3/LC3 and ACTB (loading control) immunoblots of aVICs treated with 80 nM torin-1 for 48 h in the presence or absence of 5 µM baf-A1 for 4 h (n=3). (**B**) Quantification of the tested proteins:ACTB in (**A**), normalized to aVICs treated with vehicle. (**C**) MAP1LC3/LC3-II:MAP1LC3/LC3-I ratio in aVICs normalized to cells treated with vehicle. (**D**) Representative images of CDKN1A and CDKN2A immunostaining in aVICs treated with rapamycin for 0 h, 6 h and 48 h. Scale bars: 50 µm (n=6). (**E**) Quantification of CDKN1A and CDKN2A puncta in the nucleus (nucle) and cytoplasm (cyto) of aVICs as shown in (**D**). Results are presented as mean ± SEM. ANOVA followed by Tukey's range test. (*p < 0.05; **p < 0.01; ***p < 0.001).


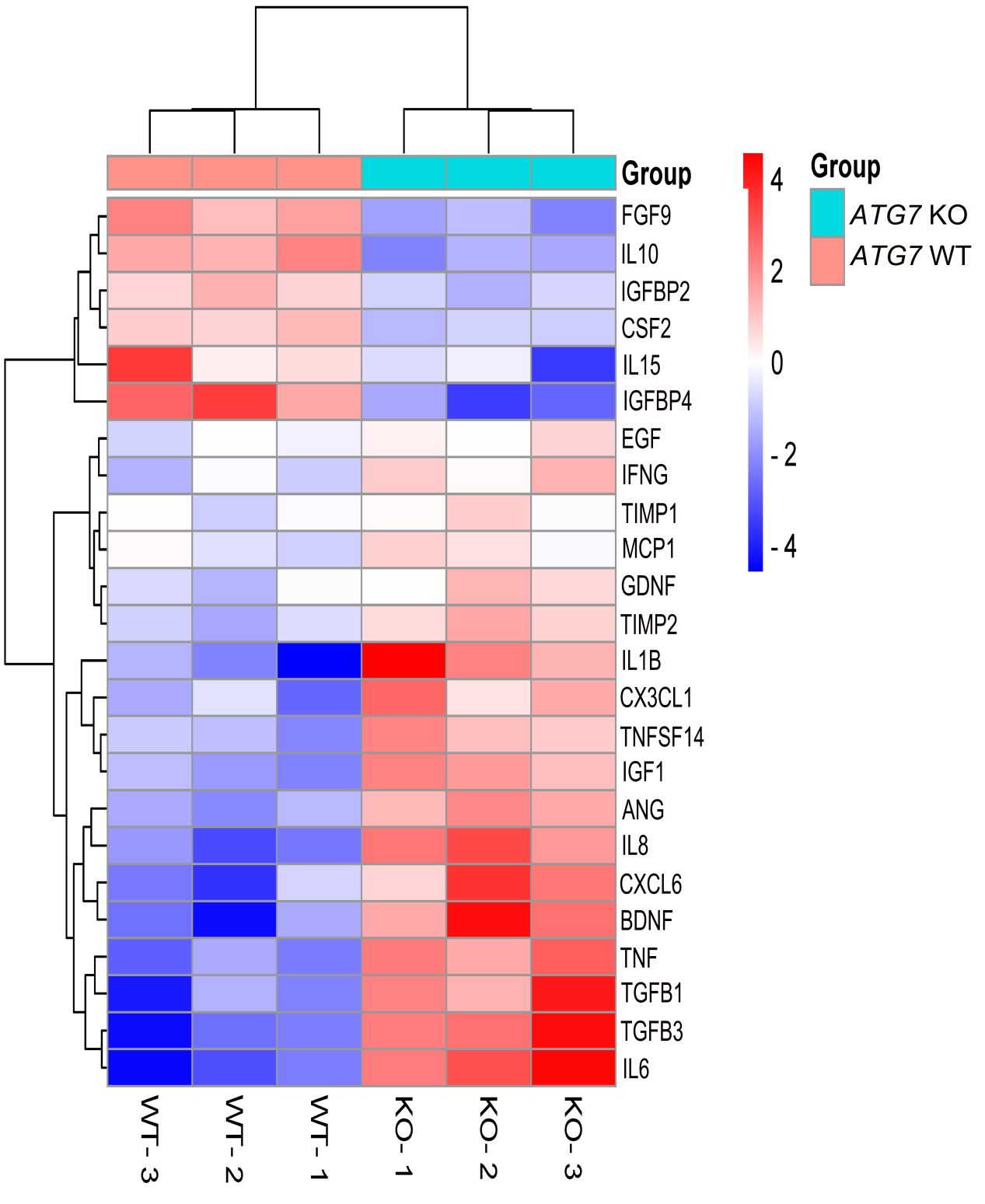


**Figure S5.** The profile of SASP factors secreted by WT and *ATG7* KO qVICs is assessed in conditioned media using a cytokine antibody array. The profile of SASP factors was assessed in conditioned media using a cytokine antibody array. The detected protein levels were normalized against internal positive controls. The data are presented as the log2 of the fold change, where fold change is calculated as the ratio of *ATG7* KO to *ATG7* WT, and visualized in a heatmap. The heatmap displays the quantification of 24 selected cytokines, with each column representing a sample and each row indicating the expression of a specific protein. A color scale was employed to represent relative cytokine expression levels, with red indicating high expression and blue representing low expression. Hierarchical clustering was visualized using dendrograms, enabling the display of the clustering structure and relationships among the data points. Data are based on three biological replicates (n=3). The evaluated proteins demonstrate a high degree of evolutionary conservation between humans and dogs, thereby supporting the rationale for employing this assay in this study.


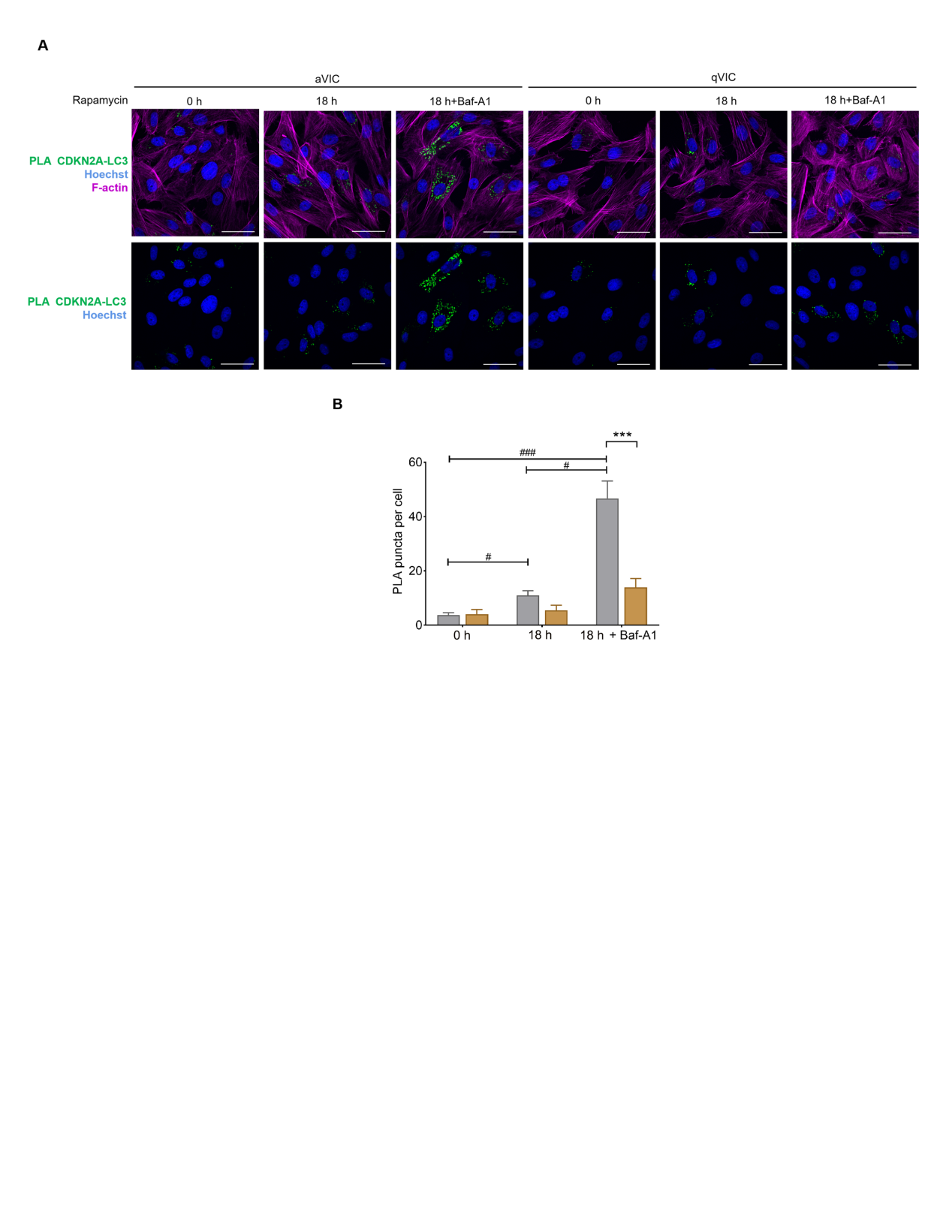


**Figure S6.** CDKN2A/p16 localizes on autolysosomes during autophagy promotion. (**A**) Representative images of PLA for CDKN2A and the autophagosome marker MAP1LC3/LC3 in aVICs treated with or without 200 nM rapamycin for 18 h in the presence or absence of 5 µM baf-A1 for 4 h (n=3). F-actin was stained with phalloidin. Scale bars: 50 µm. (**B**) Quantification of PLA puncta per cell as described in (**A**). Results are presented as mean ± SEM. ANOVA followed by Tukey's range test. (***p < 0.001; #p < 0.05; ###p < 0.001).


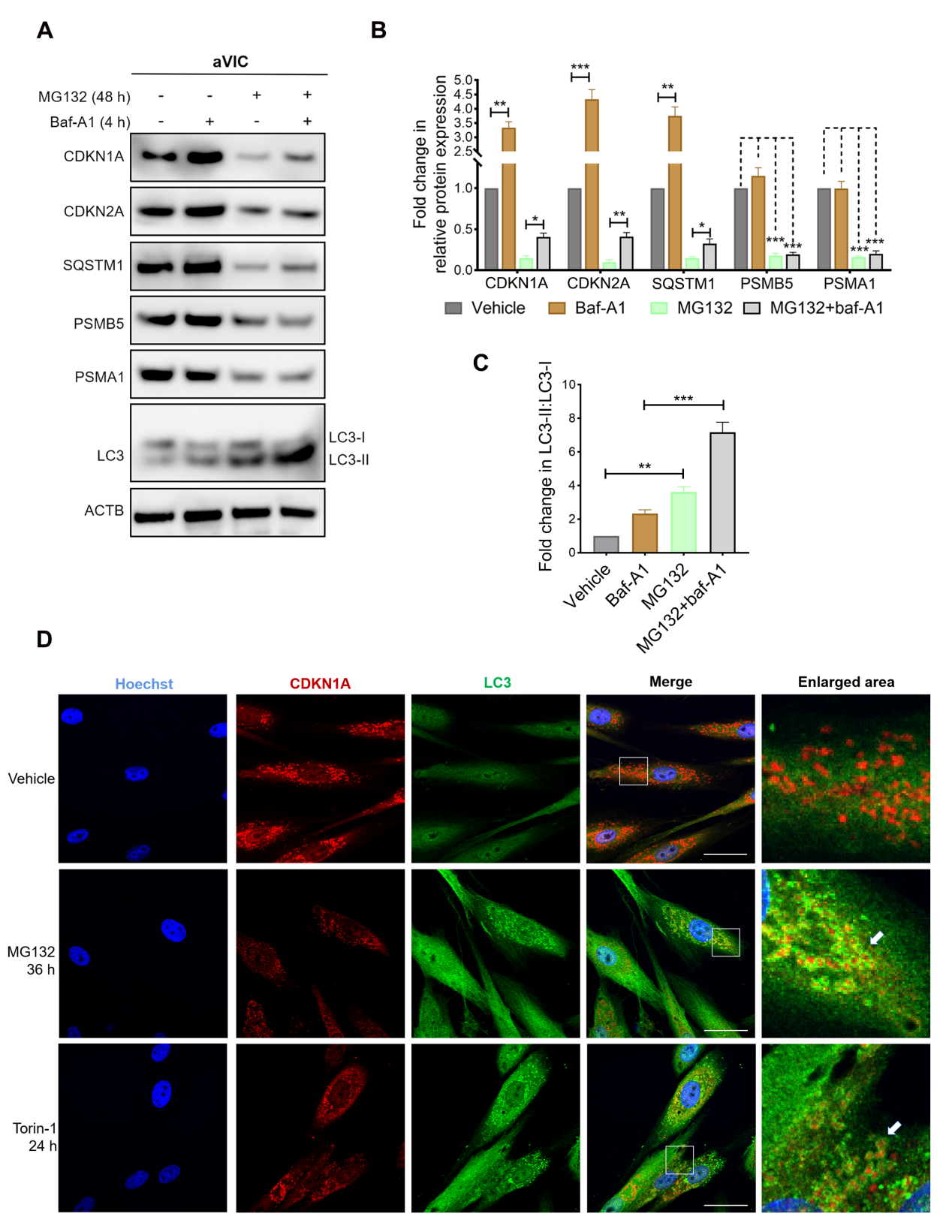


**Figure S7.** CDKN1A/p21 and CDKN2A/p16 undergo autophagic degradation during proteasomal inhibition by MG132. (**A**) Representative images of anti-SQSTM1/p62, CDKN1A, CDKN2A, PSMB5, PSMA1, MAP1LC3/LC3 and ACTB (loading control) immunoblots of aVICs treated with 10 µM MG132 for 48 h in the presence or absence of 5 µM baf-A1 for 6 h (n=5). (**B**) Quantification of the target protein:ACTB proteins, normalized to aVICs treated with vehicle DMSO. (**C**) MAP1LC3/LC3-II: MAP1LC3/LC3-I ratio in cells, normalized to aVICs treated with vehicle. (**D**) Representative high-resolution confocal images of CDKN1A colocalization with MAP1LC3/LC3 in aVICs treated with vehicle, 10 µM MG132 for 36 h or 80 nM torin-1 for 24 h. The white arrows show MAP1LC3/LC3-positive autophagosomes with sequestrated CDKN1A puncta. Scale bars: 50 µm. Results are presented as mean ± SEM. ANOVA followed by Tukey's range test. (*p < 0.05; **p < 0.01; ***p < 0.001).


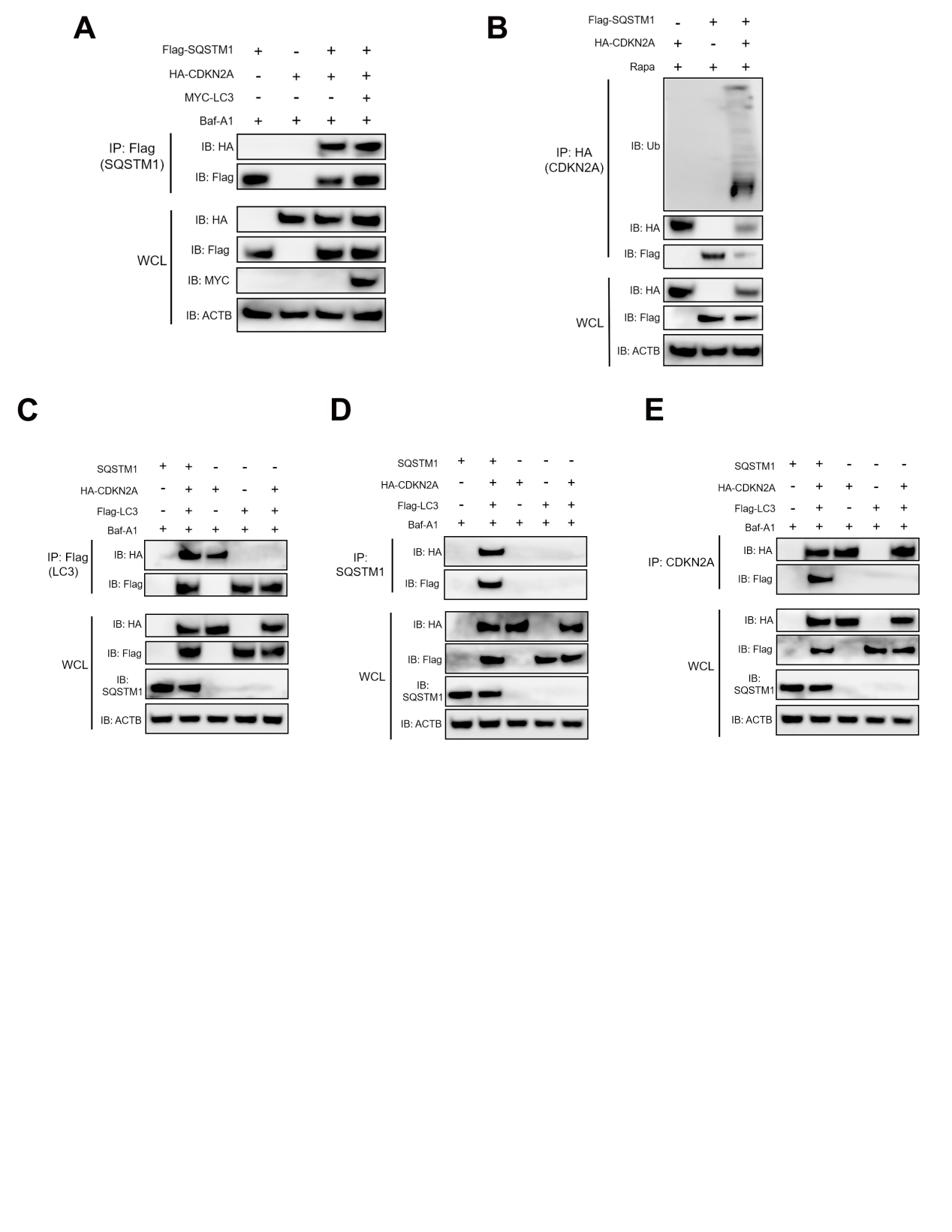


**Figure S8.** CDKN2A/p16 interacts with SQSTM1/p62. (**A**) HEK293T cells were co-transfected with *pcDNA3.1-HA-CDKN2A*, *pcDNA3.1-MYC-MAP1LC3/LC3* and *pcDNA3.1-Flag-SQSTM1* for 24 h. Flag, HA, MYC and ACTB (loading control) immunoblots for IP of Flag from HEK-293T cells in the presence of baf-A1 and the corresponding WCL (n=3). (**B**) IP of HA from aVICs co-transfected with *pcDNA3.1-HA-CDKN2A* and *pcDNA3.1-Flag-SQSTM1* in the presence of 5 µM baf-A1 for 4 h. Immunoblots for anti-HA, Flag, ubiquitin (Ub), and ACTB (n=3). (**C-E**) Three-way IP of SQSTM1, MAP1LC3/LC3, CDKN2A in HEK-293T cells. HEK293T cells were co-transfected with *pcDNA3.1-SQSTM1*, *pcDNA3.1-HA-CDKN2A* and *pcDNA3.1-Flag-MAP1LC3/LC3*. Flag, HA, SQSTM1 and ACTB immunoblots for the three-way IP of SQSTM1, MAP1LC3/LC3, CDKN2A from HEK-293T cells in the presence of baf-A1 and the corresponding whole-cell lysate (WCL) (n=2).


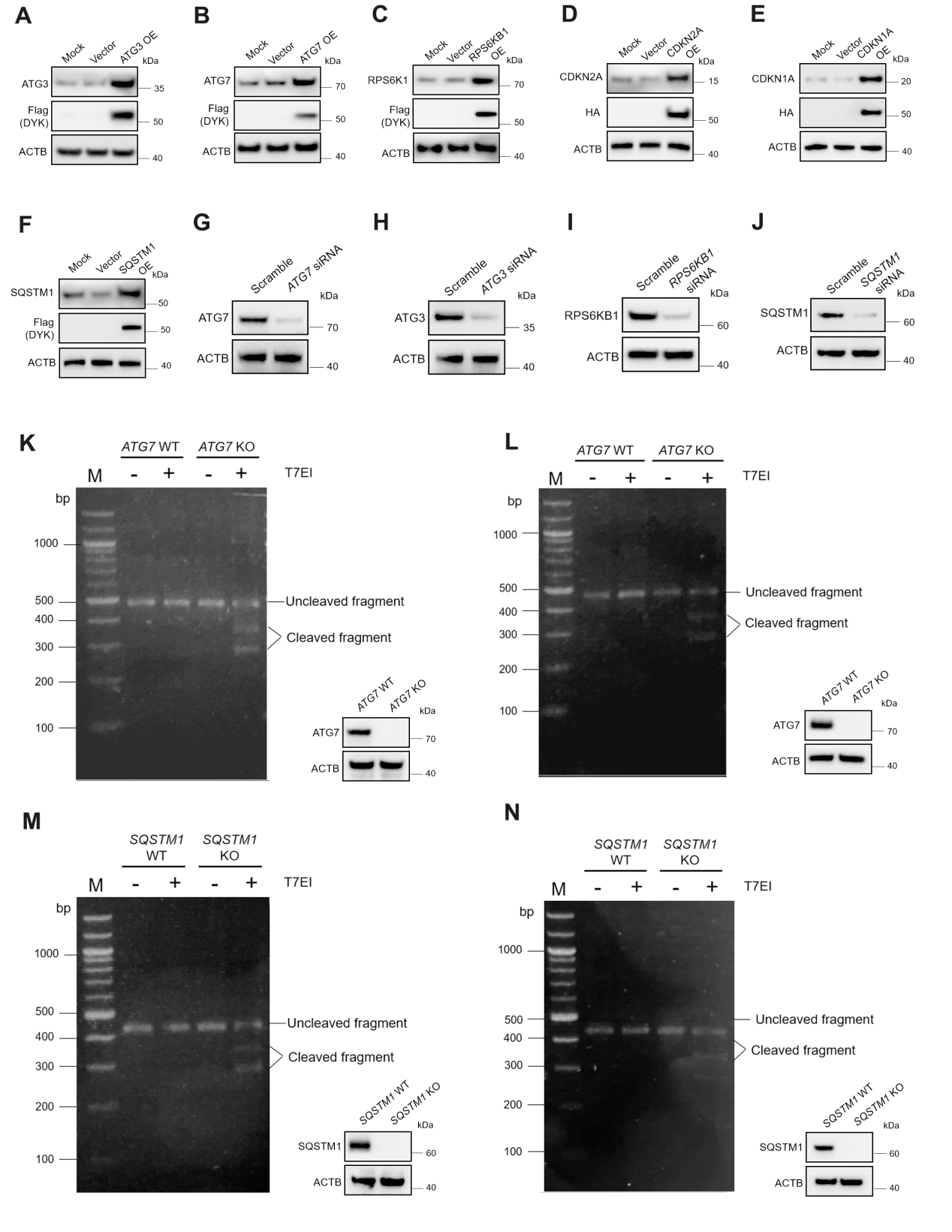


**Figure S9**. Validation for gene overexpression, siRNA knockdown and CRISPR-cas9 knockout. (**A-F**) Immunoblots for ATG3, ATG7, RPS6KB1, CDKN2A/p16, CDKN1A/p21, SQSTM1/p62, Flag (DYK), HA and ACTB (loading control) in cells transfected with empty vector, *pcDNA3.1-Flag-ATG3,* *pcDNA3.1-Flag-ATG7, pcDNA3.1-Flag-RPS6KB1, pcDNA3.1-HA-CDKN2A, pcDNA3.1-HA-CDKN1A* or *pcDNA3.1-Flag-SQSTM1*. (**G**-**J**) Immunoblots for ATG3, ATG7, RPS6KB1, SQSTM1 and ACTB in cells transfected with scramble siRNA, *ATG7*-specific siRNA, *ATG3*-specific siRNA, *RPS6KB1*-specific siRNA or *SQSTM1*-specific siRNA. (**K** and **L**) PCR products from wild-type (WT) and *ATG7* knockout (KO) VICs (**K**) and HEK293T cells (**L**) were treated with or without T7 Endonuclease I (T7EI) and then analyzed by agarose gel electrophoresis (left panel). Protein lysates were analyzed by immunoblotting (right panel). (**M** and **N**) PCR products from WT and *SQSTM1* KO VICs (**M**) and HEK293T cells (**N**) were treated with or without T7EI and then analyzed by agarose gel electrophoresis (left panel). Protein lysates were analyzed by immunoblotting (right panel).
